# Operando X‐ray Spectroscopy Study of Pd and Pd–Au Laterally Condensed Catalysts during Selective Acetylene Hydrogenation: The Role of Carbon

**DOI:** 10.1002/adma.202517227

**Published:** 2026-01-10

**Authors:** Eylül Öztuna, Thomas Götsch, Daniel Cruz, Patrick Zeller, Olga V. Vinogradova, Zehua Li, Christian Rohner, Franz‐Philipp Schmidt, Zahra Gheisari, Alexander Steigert, Martin Muske, Michael Hävecker, Eugen Stotz, Iryna Antonyshyn, Frank Girgsdies, Olaf Timpe, Thomas Lunkenbein, Vanessa J. Bukas, Karsten Reuter, Robert Schlögl, Katarzyna Skorupska, Axel Knop‐Gericke, Beatriz Roldán Cuenya

**Affiliations:** ^1^ Fritz‐Haber‐Institut der Max‐Planck‐Gesellschaft, Department of Inorganic Chemistry Faradayweg 4‐6 Berlin Germany; ^2^ Helmholtz‐Zentrum Berlin für Materialien und Energie Albert‐Einstein‐Straße 15 Berlin Germany; ^3^ Fritz‐Haber‐Institut der Max‐Planck‐Gesellschaft, Theory Department Faradayweg 4‐6 Berlin Germany; ^4^ Max‐Planck‐Institut für Chemische Energiekonversion Stiftstraße 34‐36 Mülheim an der Ruhr Germany; ^5^ Max‐Planck‐Institut für Chemische Physik fester Stoffe Nöthnitzer Straße 40 Dresden Germany; ^6^ Fritz‐Haber‐Institut der Max‐Planck‐Gesellschaft, Department of Interface Science Faradayweg 4‐6 Berlin Germany

**Keywords:** acetylene hydrogenation, operando spectroscopy, palladium‐gold, X‐ray absorption spectroscopy, X‐ray photoelectron spectroscopy

## Abstract

In this study, near ambient pressure X‐ray photoelectron and X‐ray absorption spectroscopy (NAP‐XPS and NAP‐XAS) were performed during the selective acetylene hydrogenation reaction on Pd and Pd‐Au laterally condensed catalysts. The activity and selectivity of this reaction are strongly influenced by the incorporation of carbon into the Pd and Pd–Au lattice, which is investigated by both experiment and theory. We find that Pd can take up much more carbon than Pd‐Au, which can be used to tune the activity of the catalyst during the hydrogenation reactions. The amount of the carbonaceous species dissolved in the Pd and Pd–Au lattice was quantified and their effect on the chemical and the electronic structure was tracked by means of operando spectroscopy throughout different stages of the acetylene hydrogenation reaction.

## Graphical abstract

The selective hydrogenation of acetylene over laterally condensed Pd and Pd–Au catalysts (LCCs) was investigated using operando X‐ray absorption and photoelectron spectroscopy in combination with density functional theory. Pd‐LCCs were found to incorporate significantly more carbon into the metal lattice than Pd–Au LCCs, where the carbon uptake governs the evolution of their chemical and electronic structure, exerting a pronounced influence on catalytic activity.

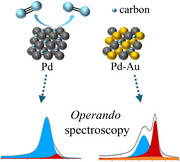

## 1 Introduction

Palladium is a highly active catalyst for selective alkyne hydrogenation reactions such as propyne [1], 1‐pentyne [2], and acetylene hydrogenation [3]. In particular, for the case of acetylene, there is high industrial and academic interest in selectively producing ethylene, primarily for purifying ethylene streams from residual acetylene, without further hydrogenating the product to the corresponding alkane, ethane. A catalyst that can eliminate trace amount of acetylene from ethylene streams is highly desirable in industry for the efficient production of polyethylene, since the presence of acetylene in the reaction feed poisons the downstream polymerization catalyst [5, 6, 7]. A selective hydrogenation catalyst would therefore need to exhibit a weaker binding of ethylene to facilitate its faster desorption from the surface and prevent the next step of the reaction towards ethane production [8]. Another promising application of selective hydrogenation of acetylene is the conversion of CO_2_ to green ethylene. In this process, the hydrocarbons as the hydrogenation products are converted to solid carbon by pyrolysis, where acetylene and ethylene are produced as side‐products. Acetylene needs to be eliminated from this system via a selective hydrogenation, as its presence creates safety issues for the downstream processes [9, 10].

Strategies for influencing the hydrogenation selectivity include the formation of a Pd‐carbon near‐surface phase (Pd:C) [3, 5], or alloying Pd with a noble metal such as gold [11]. In the former example, a Pd:C phase, located subsurface, inhibits hydrogen diffusion and prevents its participation in surface chemistry. Therefore, the amount of surface hydrogen becomes too limited for full hydrogenation to proceed to produce ethane [5]. At higher hydrogen partial pressures, a Pd‐hydride phase forms, reversing the scheme to favor the undesirable ethane production [1]. The formation of the Pd:C phase also downshifts the d‐band center, which causes weaker binding of acetylene and ethylene at the surface and leads to lower activity and higher selectivity [10]. Alloying, on the other hand, causes changes in the chemical properties via geometric (ensemble) and electronic effects [12]. Mechanistically, isolation of Pd active centers by the secondary component increases the selectivity by reducing the hydride formation [13] and by modifying the electronic structure (d‐band shift), along with a partial suppression of oligomerization, which causes catalyst deactivation [7, 14, 15]. Due to these advantages, Pd–Au has been explored for selective alkyne hydrogenation [11], alcohol oxidation [16], and water purification applications [18]. However, the rational experiment design of these systems presents practical challenges, such as how to achieve a precise control over the amount of carbon and carbon‐metal configurations in the near‐surface regions. A Pd–Au surface is also susceptible to in situ surface segregation [19], dependent on the carbon‐containing gas feed and other environmental parameters, making the exact surface structure hard to predict. The dynamic structural changes during the catalysis, i.e., segregation, agglomeration, or chemical environment evolution, pose a challenge in the operando characterization of the catalyst.

In order to gain better insight into these processes and their possible interplay, we resort to the concept of laterally condensed catalysts (LCCs), which we have recently introduced [10]. LCCs are a highly dense 2D agglomeration of nanoparticles that can be understood as quenched nucleation sites for thin films. Such material systems are highly efficient catalysts that minimize the amount of material employed, providing higher catalytic activity than their three‐dimensional counterparts (conventional supported powder catalysts) [10]. The LCC structures can be produced by well‐established thin film deposition techniques, which allow high control over the chemical and physical structures, constituting ideal systems for the exploration of surface structures and processes during the selective acetylene hydrogenation. Thus, LCCs can be utilized as both model and a functional catalytic system. Due to their 2D nature, they have also enhanced accessibility to the modern surface spectroscopic and microscopic techniques. On the contrary, conventional powdered catalysts are not easily accessible to many spectroscopic tools. Other metal systems such as single crystals are not very reactive due to the low surface area, whereas LCCs exhibit promising catalytic activity [10].

The changes that a catalyst undergoes under reaction conditions are crucial to determine which surface structures and species are the most catalytically relevant, which is key for the development of more efficient catalysts. One of the versatile methods to track these dynamic changes during catalysis is near ambient‐pressure X‐ray photoelectron spectroscopy (NAP‐XPS), allowing the investigation of the surface chemistry while the reactant gases are flowing above the catalyst surface, albeit at much lower pressures than real reactor conditions (mbar range in NAP‐XPS vs bar range in a real reactor). We have also recently developed an ambient pressure operando reaction cell which can be operated at 1 bar and where simultaneous X‐ray absorption measurements can be conducted. Thus, the pressure gap in many chemical reactions is closed by the characterization of the catalyst from the mbar to bar range. As a catalyst used in a model reaction, here we focus on a palladium LCC for selective acetylene hydrogenation, and how incorporation of carbon in the lattice is influenced by the addition of a second metal, i.e., gold. The influence of carbon incorporation on the chemical and electronic structure, as well as the catalytic activity is investigated in a large pressure range, from 1 mbar to 900 mbar, closing the pressure gap for acetylene hydrogenation. The aim of this study is thus to explore the role of the second element, i.e., Au, on the atomistic level for the acetylene hydrogenation reaction.

Here, we present operando (1 mbar) NAP‐XPS and near ambient‐pressure near‐edge X‐ray absorption fine structure (NAP‐NEXAFS) spectroscopy on Pd and Pd–Au LCCs performed under reaction conditions. In order to avoid the aforementioned ethane selectivity originating from the palladium hydrides, and to decouple carbon and hydrogen incorporation effects in the lattices (which would otherwise compete for the same interstitial sites), we performed the NAP‐XPS and NEXAFS measurements in a pressure region where the hydride phases are unstable (1 mbar) [20]. Complementarily, the electronic structure of the Pd and Pd–Au LCCs are also investigated by NEXAFS measurements at 900 mbar by using a custom‐made ambient pressure reaction cell setup. The spectroscopic measurements are supported by density functional theory (DFT) calculations. The carbon amount dissolved in the Pd and Pd–Au LCCs are quantified by combining the analysis of the NEXAFS spectra with the X‐ray Diffraction (XRD) data of Pd and Pd–Au bulk reference materials.

## 2 Results

### 2.1 NAP‐XPS during Selective Acetylene Hydrogenation: Pd

**FIGURE 1:**
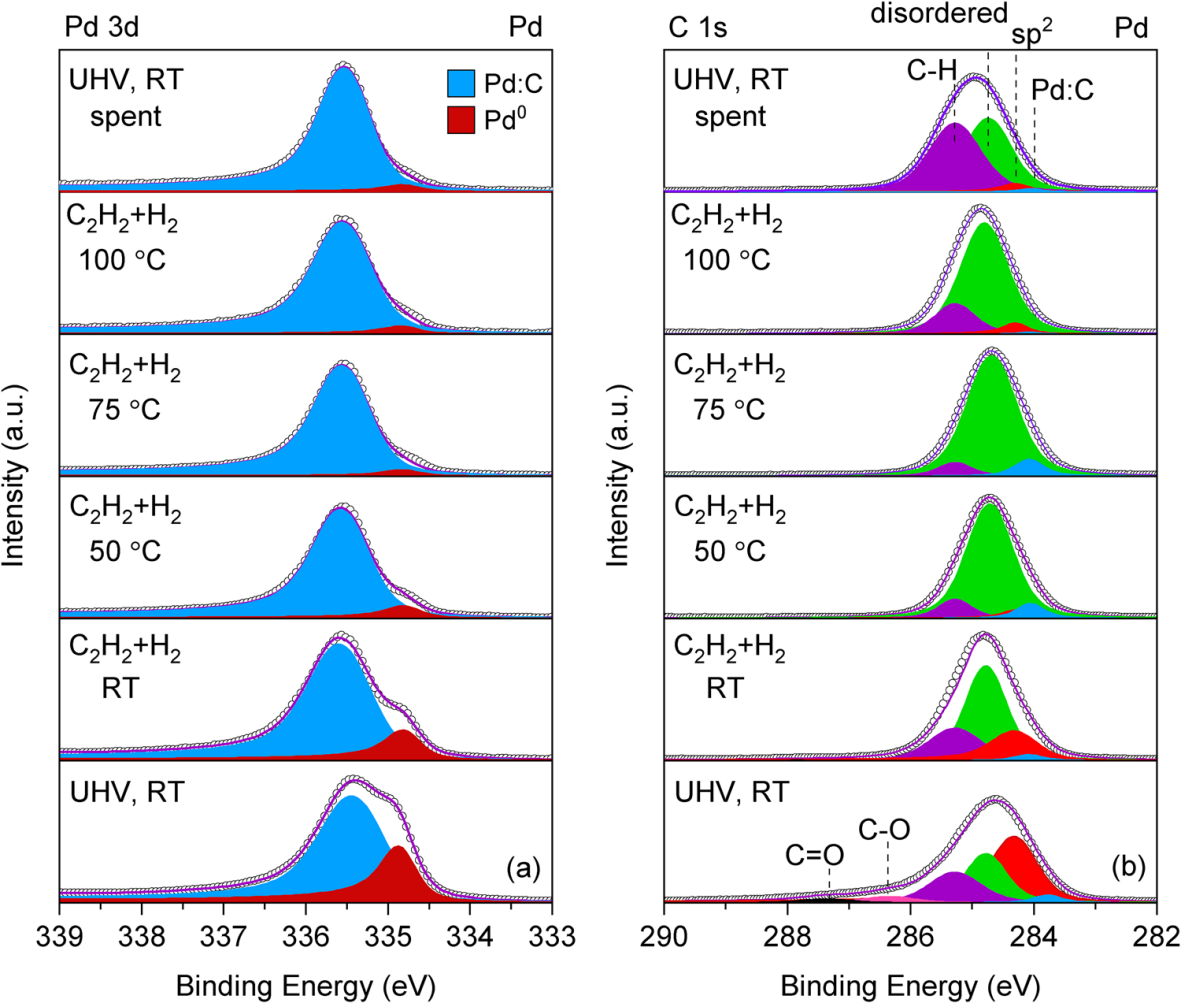
XPS (a) Pd 3d_5/2_ and (b) C 1s spectra of the Pd LCC during selective acetylene hydrogenation. C_2_H_2_:H_2_ ratio is 1:10 and the total pressure is fixed at 1 mbar. The kinetic energy of the photoelectrons is 400 eV, corresponding to an IMFP of 7 Å.

The surface structure of Pd and Pd‐Au LCCs was investigated during the selective acetylene hydrogenation. Figure 1a shows background‐corrected Pd 3d_5/2_ spectra of the Pd LCC before, during, and after the operando acetylene hydrogenation reaction. Full range Pd 3d spectra are presented in Figure S2. The Pd 3d_5/2_ spectrum in UHV and RT consists of contributions from metallic Pd and Pd with dissolved carbon (Pd:C) at 334.9 and 335.4 eV, respectively (Figure 1a; Table S1). The core level shift of the Pd:C peak is consistent with the previously reported carbon‐incorporated Pd structures [21]. The presence of surface Pd oxide or bulk Pd oxide, which would overlap with Pd:C in binding energy, can be excluded based on the Pd 3p/O 1s spectra (Figure S4). In our previous report [10], we have presented the peak positions of surface and bulk Pd oxides by in situ performed oxidation experiments on a Pd foil and Pd LCC. As the Pd 3p spectrum of Pd LCC does not show any of the oxide peaks, we have not included these species in the peak fitting of the Pd 3d spectra.

The intensity ratio between the Pd:C peak and the metallic Pd peak changes throughout the acetylene hydrogenation reaction. From Figure 1a, it can be seen that upon the introduction of the reaction mixture (acetylene:hydrogen = 1:10) to the XPS cell, there is an increase in the Pd:C to Pd metal peak ratio, which enhances further with the increase in reaction temperature. The quantification of the change in the Pd:C amount will be discussed later in the section devoted to the carbon incorporation in the Pd and the Pd–Au LCCs. Apart from the changes in the Pd:C peak intensity relative to the metallic Pd peak, there are several changes observed in the peak parameters throughout the reaction (see Table S1). First, the binding energy of the metallic Pd shifts to a lower value (by 0.1 eV) when the reaction feed is first dosed at RT. Second, the Pd:C peak shifts to higher binding energies under the reaction feed at RT, followed by a gradual shift to lower values with the increase in the reaction temperature. The binding energy difference between the Pd:C and the metallic Pd peak increases from 0.5 to a maximum of 0.8 eV (at 50°C) and lowers back to 0.7 eV (at 75°C on). After the reaction, the binding energy difference remains at 0.7 eV, which is higher than the initial value of the fresh sample before the reaction. The last change observed is the increase in the FWHM of the Pd:C peak until 50°C and its slight decrease thereafter at higher temperatures.

The presence of the Pd:C species during the reaction was observed not only in the Pd 3d, but also in the corresponding C 1s spectra. In the Pd 3d_5/2_ region, it is observed as a peak at around 335.4 eV, and in the C 1s spectrum, it is observed as a lower binding energy component at 284.1 eV, shown in Figure 1b and tabulated in Table S2. In previous operando XPS studies performed with various Pd catalysts, this low binding energy Pd:C component is observed in a range from 283.4 to 284.1 eV, depending on the alkyne/alkene utilized in the reaction feed [1, 2, 22]. For the Pd LCC, apart from the Pd:C peak at 284.1 eV, peaks for sp^2^ carbon, disordered carbon, carbon‐hydrogen (C‐H), carbonyl (C‐O), and carboxyl (C═O) species are added respectively to the deconvolution at binding energies of 284.3, 284.8, 285.3, 286.0, and 288.0 eV to account for the adventitious carbon unavoidably present on the sample initially [23, 24]. As the binding energies of all the aforementioned peaks are very close to each other, the deconvolution of C 1s core level is highly complex. Upon exposure of the sample to the reaction gas feed at RT, the higher binding energy contributions of carbonyl and carboxyl groups disappear.

The valence band (VB) spectrum of the as‐prepared Pd LCC, shown in Figure S5a (spectrum labelled as UHV, RT), features a steep onset, which indicates the Fermi edge of metallic Pd. When the reaction mixture (acetylene and hydrogen) is introduced to the system, the metallic Fermi edge remains (although the onset becomes a bit flatter), but there are some changes observed at the higher binding energies: the additional, weak bands observed around 7 and 9 eV are possibly originating from the carbonaceous deposits formed under reaction conditions [25].

### 2.2 NAP‐XPS during Selective Acetylene Hydrogenation: Pd–Au

**FIGURE 2:**
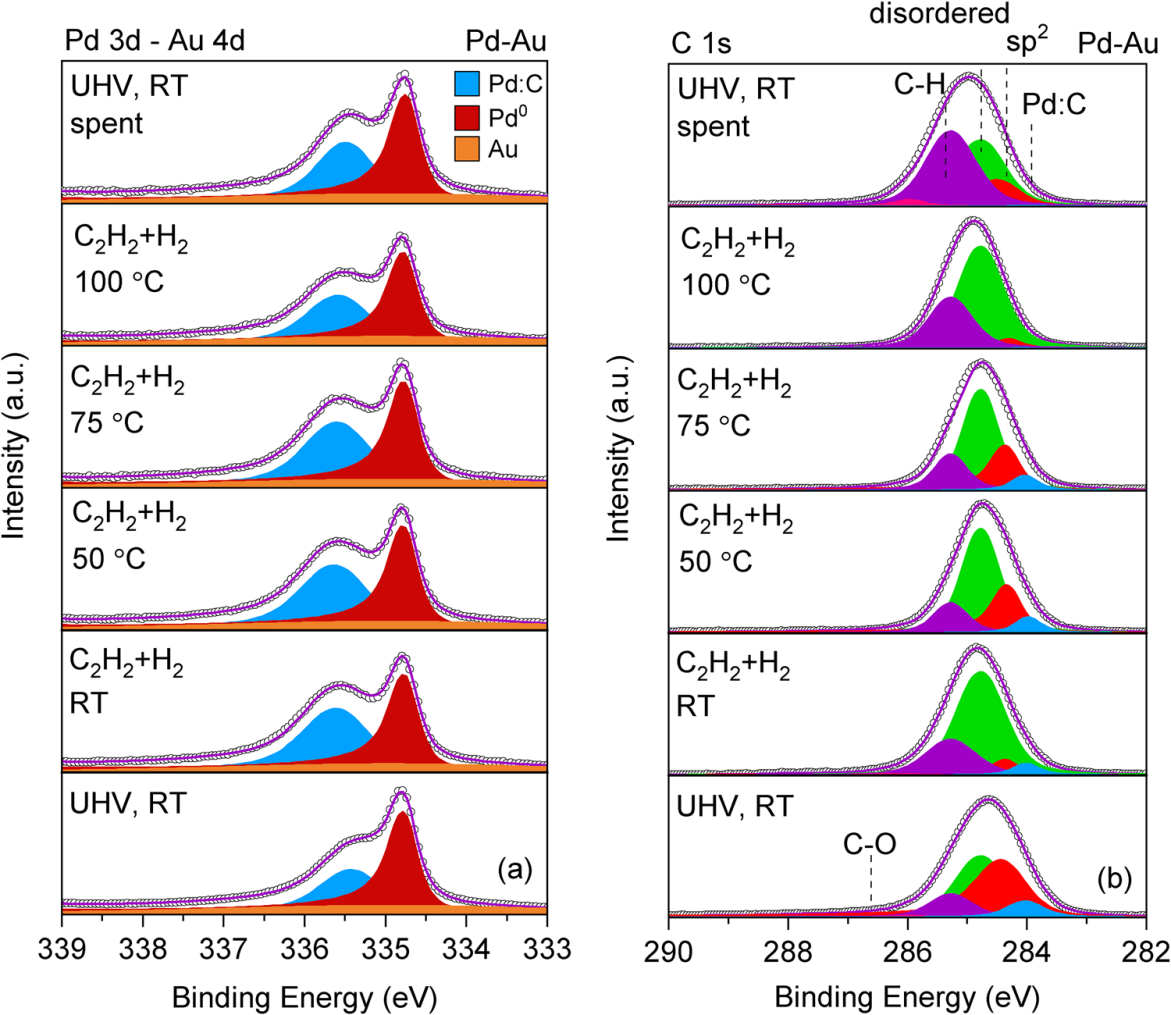
XPS (a) Pd 3d_5/2_ and (b) C 1s spectra of the Pd–Au LCC during the selective acetylene hydrogenation. C_2_H_2_:H_2_ ratio is 1:10 and the total pressure is fixed at 1 mbar. The kinetic energy of the photoelectrons is 400 eV, corresponding to an IMFP of 7 Å.

Operando XPS measurements are also performed for the Pd–Au LCC, prepared in a nominal composition of Pd:Au = 50:50. The Pd 3d_5/2_ spectrum of Pd–Au LCC consists of metallic Pd–Au, Pd:C, and the overlapping Au 4d_5/2_ peaks in UHV and RT (Figure 2a). Full range Pd 3d‐Au 4d spectra are presented in Figure S3. Like for the pure Pd LCC, no surface or bulk Pd oxide peaks are added in the fitting, since these species are not observed in the Pd 3p/O 1s spectrum (Figure S4). The metallic Pd–Au peak is observed at 334.8 eV, which is 0.1 eV lower than that of pure Pd. Shifting of the core levels toward lower binding energies compared to the pure metal counterparts is generally observed in Pd–Au alloys, and results from the gain of d electrons from Au in the case of Pd 3d [15, 26, 27]. Compared to Pd, the Pd–Au LCC can incorporate much lower amount of carbon atoms in its structure, as evidenced by the lower relative intensity of the Pd:C component to the metallic Pd–Au peak in the fresh sample (Figure 2a). When the reaction mixture of acetylene and hydrogen is introduced and the temperature is raised, there is a slight increase in the relative intensity of the Pd:C component. The Pd:C amount in Pd and Pd–Au LCCs will be discussed quantitatively in the next section describing the carbon incorporation effects. The evolution of the peak parameters for the Pd–Au LCC throughout the reaction are tabulated in Table S3. In contrast to what is observed for the Pd LCC, there is no change in the metallic Pd–Au peak binding energy under operando conditions. On the other hand, the changes in the Pd:C peak's binding energy and the FWHM values are similar to that of Pd LCC. The C 1s spectrum of the as‐prepared Pd–Au is deconvoluted into peaks assigned to Pd:C, sp^2^, disordered C, C‐H, C‐O, and C═O (shown in Figure 2b and tabulated in Table S4). The valence band spectrum of the as‐prepared Pd‐Au LCC is shown in Figure S5b and has an additional peak around 5.7 eV, which belongs to Au 5d_3/2_, showing the incorporation of Au into the structure [28]. The VB spectra during the reaction exhibit a steep Fermi edge (Figure S5b), indicating no distortion in the metallic state. The additional Au 5d_3/2_ peak also remains unchanged over the course of the reaction.

### 2.3 Carbon Incorporation in Pd and Pd–Au

**FIGURE 3:**
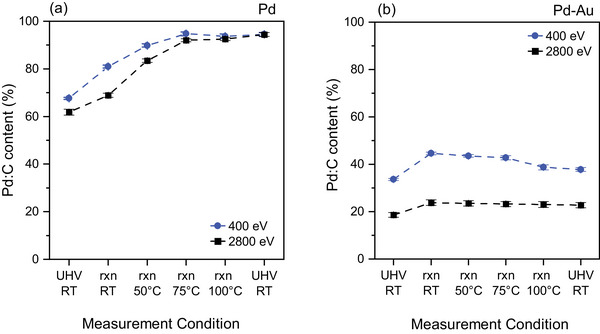
Evolution of the Pd:C content in (a) Pd and (b) Pd–Au LCC during the operando measurements, using a C_2_H_2_:H_2_ ratio of 1:10 at a total pressure of 1 mbar. Pd:C%  is calculated by the percentage of Pd:C peak area within the overall Pd 3d peak area at a kinetic energy of photoelectrons of 400 eV, corresponding to an IMFP of 7 Å. Error bars shown on the data points originate from the uncertainties in the fitting routine. 'rxn' is abbreviation for 'reaction' (C_2_H_2_+H_2_).

In the as‐prepared Pd LCC, the Pd:C component constitutes respectively 68% and 62% of the Pd 3d peak at the kinetic energy of photoelectrons of 400 (IMFP: 7 Å) and 2800 eV (IMFP: 28 Å) (Figure 3a). The higher Pd:C concentration at a kinetic energy of 400 eV compared to that of 2800 eV indicates a higher population of the Pd:C species near the surface/subsurface regions. The high amount of carbon incorporated into the Pd lattice in the fresh sample could be explained by sample preparation and transfer steps. As was reported previously [10], the Pd LCC samples were prepared in a carbon‐rich environment, which resulted in excellent catalytic activity due to the self‐repairing feature of the subsurface carbon (Pd:C) formed. The samples were thereafter transferred to the NAP‐XPS cell, after exposure to two different glovebox atmospheres, which have oxygen and moisture filters. The sample transfer steps bring additional adventitious carbon. The evolution of the Pd:C contribution for the Pd LCC throughout the reaction is demonstrated in Figure 3a for kinetic energies of 400 and 2800 eV. When the reaction mixture is introduced into the chamber and the temperature is raised, the Pd:C contribution at both kinetic energies increase gradually until they reach a saturation at around 75°C. The fact that both kinetic energies reaching to similar Pd:C concentrations indicates that the carbon is dissolved throughout the LCC structure homogeneously at higher temperatures. At the end of the run when the acetylene and hydrogen gases are evacuated from the chamber, the Pd:C content seems to increase further, which can be explained by the measurement sequence. After acquiring the Pd 3d spectrum at 100°C that is used to calculate the Pd:C content, the reaction proceeded for a while during the measurement of other core level spectra. The measurement of the Pd 3d spectrum after the reaction (spent sample) is thus performed several hours after the data quantified for 100 °C.

Unlike the Pd LCC, the Pd:C peak covers respectively 34% and 19% of the overall Pd 3d signal in the Pd–Au LCC at the kinetic energy of photoelectrons of 400 and 2800 eV (Figure 3b), which corroborates that the amount of carbon dissolved in the Pd–Au lattice is much lower than that of Pd. The Pd:C concentration increases with the exposure to the reaction mixture at room temperature at both kinetic energies, and thereafter decays gradually with the increase in reaction temperature. Even though the Pd:C concentration increases by similar amounts under the reaction conditions at room temperature for both Pd and Pd–Au, the gradual decay in its concentration at higher temperatures for the case of Pd‐Au indicates that the interaction between Pd–Au and the dissolved carbon is not strong enough to sustain the additional amount of carbon entering the lattice. It has been also previously shown in the literature that the addition of Au suppresses the carbon incorporation into the Pd lattice [29]. However, to the best of our knowledge, the lower interaction of carbon with Pd–Au alloys when compared to pure Pd counterparts and its influence on the (sub‐)surface has not been demonstrated before with XPS studies.

**FIGURE 4:**
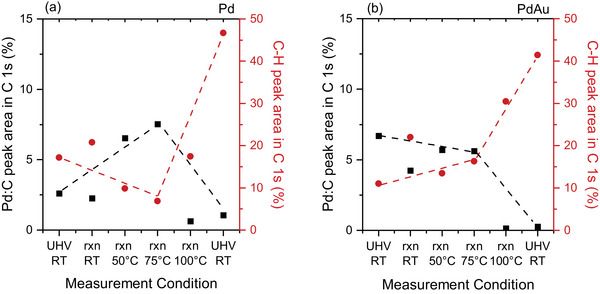
Pd:C and C–H peak area contributions (in %) to the overall peak area in the C 1s spectra of the (a) Pd and the (b) Pd‐Au LCC during the operando measurements, using a C_2_H_2_:H_2_ ratio of 1:10 at a total pressure of 1 mbar. C 1s spectra acquired at 400 eV kinetic energy of photoelectrons, corresponding to an IMFP of 7 Å. 'rxn' depicts 'reaction' (C_2_H_2_+H_2_).

Similar to the calculation performed from the Pd 3d spectra, the Pd:C content is also derived from the area of the C 1s peak and plotted for different stages of the reaction as Pd:C% (Figure 4a). The relative contribution of the Pd:C peak in the C 1s spectra increases up to 75°C, and decreases thereafter with increasing reaction temperature to 100°C and stays constant after the reaction. Simultaneously, a gradual decrease in the C–H peak area (percentage of the C–H peak area within the overall C 1s peak area) is observed up to 75°C, followed by a sudden increase at higher temperature. Further increase in the C–H area after the reaction (spent sample) is related to the measurement sequence. The C 1s spectrum is measured at the initial stages of a new measurement step (i.e., temperature change) and the reaction proceeds for a couple of hours before a new condition is applied. Thus, the reaction continues for several hours between the C 1s measured at 100°C in the reaction mixture and the UHV, RT measurement performed after the reaction. Figure 4b shows that for the Pd–Au LCC, the Pd:C content minimally changes until 75°C. Raising the temperature to 100°C in the reaction feed, however, resulted in a decrease of the Pd:C peak area, similar to what was observed for Pd at the same temperature (Figure 4b). There is also an increase in the C–H peak area at the last temperature step of 100°C during the reaction, which grows further after switching off the reaction mixture.

**FIGURE 5:**
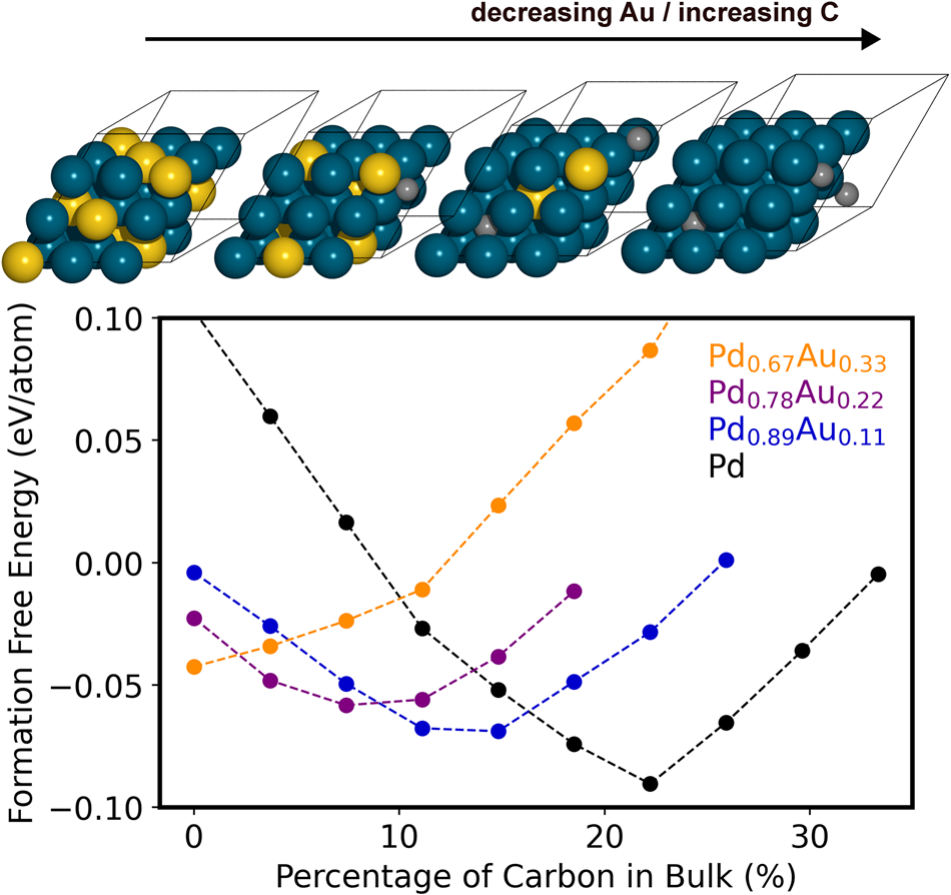
Formation free energy per atom for bulk Pd and Pd–Au alloys versus the atomic % of intercalated carbon, as calculated from DFT. Different colored markers represent a decreasing Pd:Au alloying ratio as indicated in the label. Full circles correspond to explicitly calculated DFT data, while dashed lines are meant to guide the eye. An atomic view of the predicted minimum‐energy DFT model structures is shown above (Pd:teal, Au:yellow, C:gray).

Our DFT simulations support the finding of reduced C intercalation within the Pd–Au as compared to the pure Pd lattice, in agreement also with previous work [29]. Figure 5 specifically shows the per‐atom formation free energy of bulk Pd and Pd–Au alloy structures as a function of the intercalated C in octahedral lattice sites. Free energies are calculated such that lower values represent more stable structures, while referencing metallic atoms against either bulk Pd or Au and C atoms against gaseous C_2_H_2_ at standard conditions (Table S5). This analysis clearly shows the existence of an optimal C content (minimum in each parabolic‐like curve) which shifts with the Pd:Au alloying ratio: pure Pd most favorably accommodates ca. 22 at.% C, but this fraction drops significantly with increasing fraction of the more noble Au component. We predict ca. 15 at.% C at 8:1 ratio of Pd:Au, ca. 7 at.% C at 7:2 ratio of Pd:Au, and no carbon at all within a 2:1 ratio of Pd:Au (at least within the 3× 3 ×3 unit cells simulated here). While shown here only for bulk C intercalation, this trend will undoubtedly hold also for near‐surface regions even though the Pd:Au composition is expected there to respond much more sensitively to the dynamically evolving reaction environment [19]. Moreover, similarly to what was reported in the case of hydrogen absorption and hydride formation, where hydrogen was shown to favor occupying purely Pd‐coordinated octahedral sites [30, 31], we find that the intercalated carbon is preferentially located near Pd atoms and we count only one Au–C contact in the case of Pd_0.78_Au_0.22_, which is probably due to the lack of a large enough monometallic Pd domain in our small (3x3x3) simulation cells. Note that carbon occupies octahedral sites with total six contacts to neighboring metal atoms.

### 2.4 NAP‐NEXAFS during Selective Acetylene Hydrogenation: Pd and Pd–Au

**FIGURE 6:**
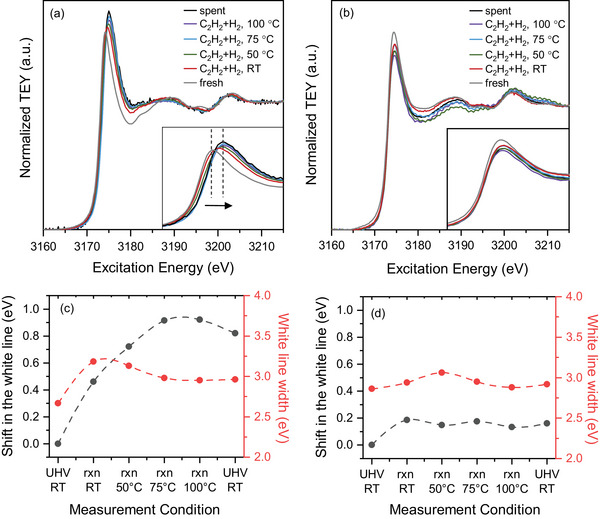
NEXAFS‐TEY Pd L_3_‐edge spectra of the (a) Pd and the (b) Pd–Au LCC during acetylene hydrogenation. C_2_H_2_:H_2_ ratio is 1:10 at a total pressure of 1 mbar. (c) and (d) quantify the white line shift (with respect to the white line energy of the as‐prepared LCC) and the white line width for Pd and Pd–Au, respectively. 'rxn' depicts 'reaction' (C_2_H_2_+H_2_).

**FIGURE 7:**
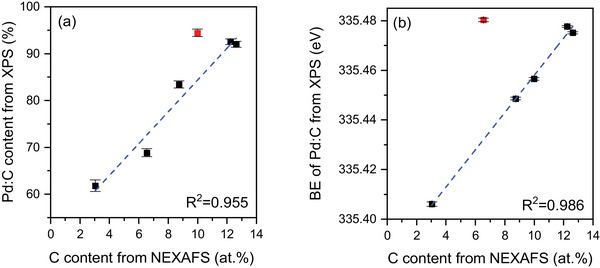
(a) Relation between Pd:C% calculated from XPS Pd 3d spectra and (b) binding energy of the Pd:C peak at 2800 eV kinetic energy vs fraction of carbon (x) in PdC_*x*_ structure calculated from the XAS analysis. The red points in (a) and (b) respectively belong to the UHV, RT, spent and rxn, RT data, and excluded before linear fitting the data. ‘rxn’ depicts ‘reaction’ (C_2_H_2_+H_2_). C_2_H_2_:H_2_ ratio is 1:10 at a total pressure of 1 mbar.

Figure 6 compares NAP‐NEXAFS Pd L_3_‐edge spectra of Pd and Pd–Au LCCs under reaction conditions, which probes the transition from 2p to 4d orbitals [32]. The Pd L_3_‐edge of fresh Pd LCC (Figure 6a) shows the expected metallic Pd features at a white line energy of 3174.2 eV [33, 34]. The white line energy is identified as the position where the maximum peak intensity is observed. When the reaction mixture (C_2_H_2_+H_2_) is dosed into the XPS cell at room temperature, the white line shifts to higher photon energies, as presented in Figure 6c. As the reaction temperature is increased further, the white line gradually shifts more to even higher energies and stabilizes at 3175.1 eV at around 75°C, equal to a positive shift in the white line energy of 0.9 eV compared to the as‐prepared sample. Along with the energy shift, there is also broadening of the white line during the reaction. Figure 6c includes the changes observed in the full width at half maximum (FWHM) of the white line throughout the reaction; it increases significantly upon first dosing of the reactants and then decreases slightly with increasing reaction temperature. These changes suggest the formation of a distinct Pd:C phase composed of carbon incorporated into the palladium lattice during the reaction [35], which have mainly been studied using hard X‐ray absorption spectroscopy on the Pd K‐edge [36, 37], although the suitability of Pd L‐edge XAS was previously suggested as well [36]. These observations are in agreement with the NAP‐XPS results, which also show an initial increase and saturation of the Pd:C amount at around 75°C, validated from both Pd 3d and C 1s spectra. When the reaction is stopped and the system is switched back to UHV, the Pd:C phase seems to be stable and does not convert back to metallic Pd. Apart from the changes in the white line position and its width, there is also a clear shift in the peak energy of the scattering path at approximately 3203 eV toward lower values during the reaction (see Figure S6a and the experimental section for the details on how the peak maximum is located for the analysis). A similar negative shift in the same peak has been previously observed upon alloying Pd membranes with Ag and is attributed to the lattice expansion in Pd [38]. In order to quantify the lattice expansion in the Pd LCC, we have utilized Pd and Pd–Au bulk reference samples in well‐defined Pd:Au ratios (see the experimental section for further details) for calibration. Figure S8a shows the measured lattice constants of the Pd–Au bulk samples in various Pd:Au ratios, which increase with the amount of Au in the sample, following Vegard's law [39]. Moreover, NEXAFS Pd L_3_‐edge spectra of the bulk Pd–Au reference samples are measured and they also show a negative shift in the peak at ca. 3203 eV with increasing Au amount, analogous to what is observed in the Pd LCC during the reaction (Figures S7b and S6a.) By using the linear relation between the lattice constants measured by XRD and the peak shift of the scattering signal in the Pd L_3_‐edge of the bulk samples (Figure S8b), the lattice constant of the Pd LCC before, during, and after the acetylene hydrogenation reaction can be extracted (Figure S10a). The lattice constant of the fresh Pd LCC is calculated as 3.91 Å, slightly higher than the measured Pd bulk reference, which might be due to the higher carbon content in the Pd LCC compared to the bulk Pd reference (Figure S10a) [40]. In order to validate the accuracy of the lattice constant calculation from XAS analysis, we also applied the same method to a carbon‐free Pd foil measured in 0.1 mbar O_2_ and at 600°C (spectrum shown in Figure S9) and calculated the lattice constant as 3.93 Å, which is in close agreement with the literature when one takes the thermal expansion of the Pd lattice into account (leading to 3.92 Å at 600°C) [41]. Furthermore, the lattice expansion of the Pd LCC (with respect to the initial as‐prepared LCC) is calculated based on the XAS peak shift at around 3203 eV and plotted in Figure S10b. Based on the lattice expansion, the amount of carbon dissolved in the Pd lattice can be derived by using an equation provided by Guo et al. [42] (see experimental section for further details). Both the lattice expansion and the carbon amount follow the same trend: they increase gradually until a reaction temperature of 75°C, followed by a slight decrease at higher temperatures. After the reaction is completed (spent sample), the lattice remains expanded, corresponding to 10 at.% carbon, and does not contract back to the as‐prepared state, in agreement with the NAP‐XPS results in 1 mbar. The maximum intercalated carbon amount and the corresponding lattice expansion quantified during the 1 mbar operando measurements (13 at.% carbon and 1.7 % lattice expansion) are also in line with the DFT predictions which suggest that 11 at.% carbon will expand the lattice by 1.8% (see Table S7). Last, we compare the effect of carbon incorporation on the XPS and XAS quantification. Figure 7a,b show linear correlation between the Pd:C concentration quantified from the Pd 3d spectra and the Pd:C peak binding energy with the carbon amount quantified from the XAS analysis. Both Pd:C% and Pd:C binding energy values are extracted from the spectra acquired at 2800 eV kinetic energy, as their depth of information is closer to the XAS measurements rather than the more surface‐sensitive measurement acquired at 400 eV kinetic energy. In Figure 7a, the spent data point (measured in UHV after the reaction) shows a hysteresis that deviates from the linear behavior and thus had to be excluded for the linear fit, probably due to the decrease in the quantified carbon amount after the reaction. On the other hand, the data acquired at RT under reaction conditions appears to be an outlier in Figure 7b, as the binding energy shift in the Pd:C at this point reached a maximum when the carbon incorporation was still very dynamic.

In contrast to the spectral changes observed in the Pd LCC, there is only a slight shift in the white line of the Pd‐Au LCC when the reaction mixture is introduced at room temperature, and it stays almost constant at higher temperatures (Figure 6b,d). The changes in the FWHM of the white line are also not as pronounced as in the case of the Pd LCC. The lattice constant of the Pd–Au LCC, calculated from the XAS peak at around 3202 eV, is shown to be 3.99 Å, which matches with a Pd to Au ratio of 50:50 (Figures S6b and S8a). Thus, the composition of the Pd‐Au LCC calculated from the XAS analysis is in good agreement with both the nominal composition and the actual composition measured by ICP‐OES (see Table S11). During the reaction, no significant shifts in the peak at ca. 3202 eV are observed for the Pd–Au LCC, unlike the pure Pd (Figure S6b). These observations further validate the suppression of carbon incorporation into the Pd–Au lattice, as demonstrated previously by the XPS results. Similar observations were also made for the hydrogen uptake in Pd alloys [30, 31]. Another notable point is the absence of a Pd hydride peak in both samples, which is typically visible distinctly as a peak in the Pd L_3_‐edge spectrum at an excitation energy around 3180 eV, derived from new antibonding states formed between Pd and H [35]. The absence of this peak validates our prior analysis of the Pd and Pd‐Au spectra by only considering the presence of dissolved carbon and not in the presence of a hydrogen amount sufficient to form Pd hydrides. This is however expected, since the hydrogen partial pressure (slighly lower than 1 mbar) is not sufficient to induce the transition from metallic Pd into PdH_*x*_ species [43].

**FIGURE 8:**
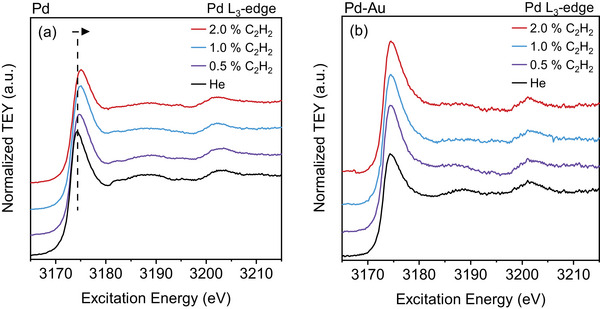
NEXAFS Pd L_3_‐edge spectra of the (a) Pd and the (b) Pd–Au LCC during the acetylene hydrogenation. C_2_H_2_:H_2_ ratio is 1:10 and balanced with He to 900 mbar. C_2_H_2_ concentration is sequentially changed from 0.5 to 2.0%. Temperature is 25°C in spectrum labelled 'He' and 100°C during the reaction.

To complement the NAP‐XPS and NEXAFS studies performed at 1 mbar in Pd hydride‐free conditions, acetylene hydrogenation is also performed at 900 mbar by using a custom‐made ambient pressure reaction cell, shown in Figure S1. As this is a flow cell with a SiN_*x*_ membrane lid, photoelectrons cannot escape, i.e., only XAS measurements can be performed. Furthermore, a direct repetition of the 1 mbar NAP‐XPS experiments at higher pressures was prevented by carbon build‐up: when introducing acetylene and hydrogen at room temperature, the higher acetylene partial pressure at 900 mbar as compared to the mbar conditions led to severe carbon deposition on the surface, attenuating the Pd L_3_‐edge signal in this surface‐sensitive measurement mode to such an extent that any spectral evaluation was impossible. Thus, the reaction is performed only at 100°C, where the carbon deposition was milder due to higher conversion of the reactant molecules. The experiment involved an initial reduction step at 125°C (in 30% H_2_ in He), followed by lowering the temperature to 100°C and introducing an acetylene and hydrogen mixture. The concentration of acetylene is changed gradually from 0.5 to 2.0%. In Figure 8a, the Pd L_3_‐edge spectra of the Pd LCC at 900 mbar reaction conditions are shown. The spectrum measured in He for the fresh sample matches with that of metallic palladium. In the reaction mixture (acetylene concentration of 0.5%), the white line shifts to higher energy, corresponding to carbon incorporation into the lattice. Consequently, the white line shift increases when the acetylene concentration is increased to 1.0 and subsequently to 2.0%. The overall shift in the white line is slightly lower at 900 mbar compared to 1 mbar conditions (at 100°C, 0.9 vs 0.7 eV), which might be due to either shorter reaction time spent at 900 mbar, or that the reaction conducted at lower temperatures at 1 mbar facilitated more carbon incorporation in the lattice compared to the introduction of the feed at 100°C. Nevertheless, similar spectral changes are observed, indicating Pd:C phase formation from the mbar to the bar range. Moreover, the lattice expansion and the carbon amount are quantified from the peak shift at ca. 3203 eV in the Pd L_3_‐edge spectra for the ambient pressure experiment (Figures S11a and S12). The magnitude of the increase in both the lattice expansion and the carbon amount are higher while increasing the acetylene concentration from 0.5 to 1.0% compared to the change from 1.0 to 2.0%, suggesting that carbon incorporation into the Pd lattice based on the acetylene partial pressure is a self‐limiting process. For the Pd–Au LCC shown in Figure 8b, there is no change in the white line position for any of the acetylene concentrations dosed, indicating that the distinct Pd:C phase formation is also not observed at elevated pressures. There is also no significant shift in the peak at ca. 3203 eV during the reaction at 900 mbar, showing the absence of a lattice expansion (Figure S11b). Both results are in excellent agreement with the measurements performed at 1 mbar. Thus, Pd:C phase formation follows the same trend from the mbar to the bar range, demonstrating the absence of a pressure gap for this particular process. Another control experiment is also performed to investigate the dynamics of hydrogen and carbon incorporation in palladium (Figure S13). When hydrogen is dosed at RT to a fresh Pd LCC, the white line position increases by 0.5 eV, and a distinct Pd hydride peak emerges at 3180 eV [35]. The increase in the temperature to 125°C results in a shift of the white line to lower energies and the hydride peak disappearing almost completely. Following this, acetylene hydrogenation is carried out at the highest acetylene concentration (2.0%) and a temperature of 100°C, leading to a peak shift (to higher energies), corresponding to Pd:C phase formation. After the reaction, the temperature is lowered to 40°C and hydrogen is introduced into the reaction cell. The white line position remains almost unchanged and no hydride peak is observed. This suggests that once the Pd:C phase is formed, the carbon cannot be removed from the Pd lattice by hydrogen incorporation.This is in agreement with the studies by Bugaev et al. [36] who observed using Pd K‐edge spectroscopy that the bulk Pd:C phases cannot be removed by hydrogen treatment. Due to the SiN_*x*_ window, the 900 mbar reaction cell only allowed XAS measurements and not XPS. The latter could only be performed before and after the 900 mbar experiments. Figure S14 shows the comparison of Pd 3d spectra of the fresh and spent Pd LCC samples. The measurements were performed by using the tender beam at 5000 eV, as the spent sample is covered with a relatively thick layer of carbon and could not be analyzed by lower kinetic energies. The fresh sample contains 43% of Pd:C concent based on the overall Pd 3d peak. After the reaction, the Pd:C concentration is increased to 84%, similar to the spent sample after the 1 mbar experiments. Thus, the structure of Pd LCC changes similarly during acetylene hydrogenation performed at 1 mbar and 900 mbar. XPS measurements could not be performed for the spent Pd–Au LCC, as the surface was completely covered with carbon and no Pd 3d signal could be acquired.

### 2.5 Surface Segregation Effects

**FIGURE 9:**
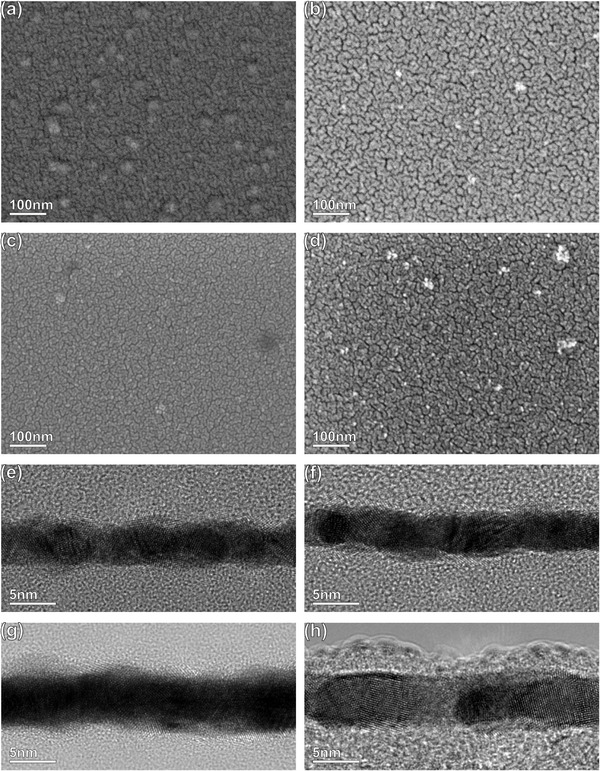
SEM images of as‐prepared Pd (a), Pd–Au (c) LCC and after the operando XPS/XAS measurements performed at 1 mbar (acetylene+hydrogen) (b,d). HRTEM cross‐section micrographs of (e,g) as‐prepared Pd–Au and after the operando XPS/XAS measurements performed at 1 mbar (acetylene+hydrogen) (f) and after the operando XAS measurements performed at 900 mbar (acetylene+hydrogen) (h).

The quantification of the Pd:Au ratio of the Pd–Au LCC from the analysis of Pd 3d‐Au 4d and Pd 3d‐Au 4f core level sets is presented in Figure S15 for two different kinetic energies of photoelectrons. The inelastic mean free path (IMFP) values for Pd–Au at 400 and 2800 eV kinetic energy are 7 and 28 Å, respectively. As the photon flux is an additional parameter for quantification in the case of the Pd 3d‐Au 4f core levels, the quantification from Pd 3d‐Au 4d, measured using the same photon energy due to their proximity, provides more accurate results. For more bulk‐sensitive measurements at kinetic energies of 2800 eV, the Pd:Au ratio is calculated as 60:40 (from both Pd 3d‐Au 4d and Pd 3d‐Au 4f), and remains unchanged during the reaction (Figure S15). The Pd content calculated from the XPS analysis in the bulk‐sensitive region is slightly higher than what is measured by ICP‐OES, (Table S11). For the more surface‐sensitive measurements at 400 eV kinetic energy, the Pd:Au ratio is found to be increasing slightly with the increase in reaction temperature (max. 65:35 at.% at 75°C). However, it cannot be concluded that there is a significant change in the surface composition throughout the reaction, considering the large error margins of the photoionization cross‐sections (Figure S15b). Previous theory studies of Pd‐Au alloys exposed to different atmospheres also reported Pd surface segregation in the presence of excess carbon in the environment [19]. Considering the high amount of carbon present already in the as‐prepared form of the samples, it can be anticipated that the segregation of Pd to the Pd–Au surface already takes place during the deposition, even before exposing the surface to higher concentrations of carbon (acetylene feed), with no further Pd segregation being detected in the time frame of these operando experiments. To validate the absence of Pd surface segregation during the operando measurements, cross‐sectional STEM measurements are performed by quantifying the Pd/Au content in different layers across the Pd–Au LCC via EDS and calculating the Pd:Au ratio. No apparent Pd segregation is observed when comparing the fresh Pd–Au sample with the spent ones after the operando XPS/XAS measurements performed at 1 mbar (Figure S16) and after the operando XAS measurements performed at 900 mbar (Figure S17). On the contrary, in the catalytic tests performed at higher temperatures (150°C) and longer durations of reaction (300 h) than in this study, slight Pd segregation to the surface is observed in the spent samples by cross‐sectional TEM analysis [40]. The enhancement of Pd on the surface might be caused by either the higher reaction temperature or the longer duration of reaction. We also have not observed any apparent dewetting or particle agglomeration effects in either Pd or Pd–Au LCC, as demonstrated by the SEM images comparing the fresh and spent samples after operando measurements performed at 1 mbar in Figure 9a–d. Additionally, no apparent particle size increase was observed in the cross‐sectional HRTEM analysis of the Pd–Au LCC after the operando measurements performed at 1 mbar (Figure 9e,f) and 900 mbar (Figure 9g,h; Figure S18). Therefore, we conclude that both Pd and Pd–Au LCC samples are analyzed mainly based on carbon incorporation effects in a large pressure range of 1 to 900 mbar, and the spectral changes we observed throughout the operando acetylene hydrogenation reaction are influenced mostly by electronic effects and not due to geometric effects such as agglomeration or segregation.

### 2.6 Catalytic Activity

**FIGURE 10:**
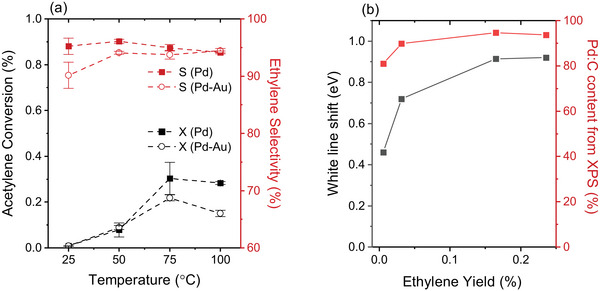
(a) Catalytic activity of Pd and Pd–Au LCCs based on gas chromatography data obtained under operando conditions during acetylene hydrogenation. C_2_H_2_:H_2_ ratio is 1:10 and the total pressure is fixed at 1 mbar. (b) Shift in the white line in NEXAFS Pd L_3_‐edge and the Pd:C amount calculated from XPS for the Pd LCC as a function of the ethylene yield. The shift in the white line is calculated with respect to the white line position of the fresh sample.

Figure 10a presents the comparison of the conversion of acetylene and selectivity of ethylene production for both Pd and Pd–Au LCCs during the operando NAP‐XPS experiments at 1 mbar. There is a trend of increasing conversion along with the increase in reaction temperature. Along with this, both samples exhibit high selectivity above 90% toward ethylene production. The difference between the selectivity values of ethylene for Pd and Pd‐Au LCCs might be insignificant compared to the error range of the GC measurement at this low conversion region. In order to complement this measurement, 1 mbar activity measurements are also performed in a conventional plug‐flow fixed‐bed reactor (Figure S20). Increasing the reaction temperature results in increasing acetylene conversion for both Pd and Pd–Au LCCs, while the selectivity values remain similar to each other and above 90%. From the data in Figure 10a, the yield of ethylene is calculated (Yield = conversion × selectivity) and plotted against the shifts in the white line in the Pd L_3_‐edge spectra and the Pd:C concentration in the XPS spectra (Figure 10b). Both, the white line shift, where a higher value indicates higher Pd:C content, and the Pd:C content increase results in higher yields of ethylene. We have also performed offline activity measurements at 900 mbar in the beamline setup for different reaction temperatures to complement the 1 mbar experiment (Figure S21). At 900 mbar, both Pd and Pd–Au LCCs show conversion increase and selectivity decrease with increasing reaction temperature.

In comparison with the offline activity measurements performed at 900 mbar in the XAS setup (Figure S21), the temperature‐dependent catalytic activity and selectivity of both Pd and Pd–Au LCCs were also evaluated at 1 bar using a plug‐flow fixed‐bed reactor. As shown in Figure S22a, the Pd LCC consistently exhibits higher acetylene conversion than the Pd–Au LCC across the entire temperature range, which can be attributed to its larger number of accessible Pd surface sites. As the temperature increases, the reaction rate rises because a greater fraction of molecules can overcome the activation energy barrier, leading to progressively higher conversion for both catalysts. The Pd LCC also shows higher selectivity toward C_2_H_4_ ‐ the desired semi‐hydrogenation product ‐ than the Pd–Au LCC at all temperatures examined. This enhanced selectivity can be rationalized by the more substantial Pd:C accumulation (atomic carbon dissolved into the Pd lattice) observed for the Pd LCC relative to the Pd–Au LCC, as indicated earlier by the NAP‐XPS and NEXAFS analyses. Atomic carbon occupying octahedral voids in the Pd lattice appears to partially assume the ensemble and electronic effects normally provided by Au when it substitutes Pd atoms in the FCC lattice, thereby functionally mimicking the selectivity‐modifying influence of Au. As shown in Figure S22c, butane formation is most pronounced at lower temperatures and decreases steadily with increasing temperature. This reflects the typical trade‐off between activity and selectivity: lower temperatures suppress full hydrogenation but promote C–C coupling, whereas higher temperatures increase overall activity but favor deeper hydrogenation pathways. Although ethylene binding weakens with increasing temperature, the intrinsic rates of both C_2_H_2_ and C_2_H_4_ hydrogenation increase even more strongly. As a result, deep hydrogenation becomes competitive before ethylene can desorb, leading to a net decrease in C_2_H_4_ selectivity at higher temperatures. For determination of the apparent activation energy, data obtained at very low conversion (near zero at 30°C) and at very high conversion (approaching diffusion‐limited behavior at 125°C) were excluded. The Arrhenius plots in Figure S22b yield apparent activation energies of 35.98 and 36.19 kJ mol^−1^ for the Pd and Pd–Au LCCs, respectively, values consistent with previously reported activation energies for Pd‐based catalysts in acetylene hydrogenation [44, 45].

Figure S23 shows the activity comparison of Pd and Pd–Au LCCs measured operando during acetylene hydrogenation performed at 900 mbar with the ambient pressure reaction cell. For the corresponding XAS spectra, refer to Figure 8. At similar conversion values, the selectivity for ethylene production is slightly higher for Pd at 0.5% acetylene (around 5%) than for Pd–Au, and the difference in selectivity between Pd and Pd–Au increases to 7% when the acetylene concentration increases to 1.0%, and does not change significantly at 2.0% acetylene.

## 3 Discussion

NAP‐XPS reveals significant changes to the electronic structure of Pd during acetylene hydrogenation. The positive binding energy shifts in the Pd:C peak upon exposure of the LCC samples to the acetylene/hydrogen feed can be explained by different core level shifts (CLS) observed upon carbon inclusion in Pd, as reported previously by DFT calculations on the metastable palladium carbide structure [46]. According to that study, Pd exhibits CLS in a wide energy range from 0.40 to 1.15 eV in the presence of carbon (and hydrogen), depending on the position of the carbon atom in the vicinity of the Pd atom, i.e., whether it is located at the surface, subsurface, or deeper layers of Pd. Thus, the electronic structure of Pd changes with the coordination of the C atoms. The negative binding energy shifts in the metallic Pd peak could also be explained by similar phenomena, i.e., some Pd atoms have been shown to exhibit a CLS of ‐0.25 eV depending on their relative position to the C atoms in the same DFT study by Seriani et al. [46]. As we observe a clear change in the binding energies when the reaction feed is first dosed and as the temperature is raised during the reaction, it is plausible that there are different Pd:C species present in the as‐prepared LCC and throughout the selective acetylene hydrogenation reaction. Aside from chemically/electronically different Pd:C species, the change in the Pd:C binding energy might be also related to the amount of the carbon incorporated into the Pd lattice. Based on XPS analysis of Pd carbide structures with different Pd/C ratios, it was previously demonstrated by Guo et al. [42] that the binding energy of the Pd:C peak gradually shifts to higher values with increasing carbon content. This, together with the linear correlation between the amount of dissolved carbon (from NEXAFS) and the binding energy of the Pd:C peak, as well as the Pd:C concentration (both from XPS) that was established earlier, suggests that the amount of carbon would play a more significant role than the nature of the Pd:C species. However, the effect of Pd:C species transforming dynamically under the reaction conditions on the observed spectral changes cannot be excluded, although the differentiation and identification of distinct Pd:C species in this homogeneous distribution is experimentally very challenging and requires further studies on the topic.

Previously, our group reported on the in‐situ characterization of various Pd structures during selective alkyne hydrogenation [1, 3]. In these studies, the active species was found to be subsurface Pd:C, formed by the diffusion of carbon into the interstitial sites of the Pd lattice. Also, the correlation between the Pd:C concentration and the selectivity toward the partial hydrogenation reaction was presented in detail. In the present work, the increase of the Pd:C phase inhibits the diffusion of highly active hydrogen species through Pd and prevents the formation of the Pd hydride phase, resulting in high selectivity toward ethylene (i.e., the desired product). Conversely, when the Pd:C amount is lower (or, alternatively, the hydrogen partial pressure is increased), then the Pd hydride becomes the dominant species, and the reaction becomes unselective.

The catalytic activity is also intricately linked to the electronic structure: for the Pd LCC, the activity increase (or conversion of acetylene) observed in the operando GC measurements is accompanied by a gradual increase of the Pd:C component in the NAP‐XPS Pd 3d spectra (at 1 mbar). Moreover, there is an increased contribution of Pd:C in Pd as compared to Pd–Au. The decrease of the carbon content with the addition of Au into the Pd lattice under reaction conditions is also demonstrated clearly in the Pd L_3_‐edge spectra, where shifts in the white line indicating the Pd:C phase formation along with the lattice expansion are observed in the pure Pd LCC but not in the Pd–Au alloy. The yield of ethylene is found to be enhanced by the increasing contribution of the Pd:C content, as corroborated by the results of XPS and NEXAFS in Figure 10b. The measurements performed at 900 mbar using the custom‐made ambient pressure reaction cell give clear hints on the role of carbon for ethylene selectivity: while only a very small (almost negligible) difference in selectivities between Pd and Pd–Au was observed under mbar conditions, the difference is significant when the pressure is increased to 900 mbar. As shown in Figure S23, at 0.5% C_2_H_2_ concentration, the selectivity for ethylene is slightly higher in Pd compared to Pd–Au. Along with this, there is a slight shift in the Pd L_3_ white line towards higher energies as well as the expansion of the lattice, as extracted from the analysis of the Pd L_3_‐edge spectra, indicating Pd:C phase formation (Figures 8a; Figure S12). Thus, the formation of the Pd:C phase in Pd results in the inhibition of H diffusion, which leads to a higher selectivity for ethylene. As the concentration of acetylene increases to 1.0%, the shift in the white line becomes more prominent, and so does the difference between the selectivity values of Pd and Pd–Au, validating the correlation between Pd:C formation and ethylene selectivity. The Pd:C phase causes weaker surface binding of ethylene and acetylene molecules, therefore leading to a high selectivity and low activity, respectively. The slightly lower activity of Pd compared to Pd–Au (lower conversion) is also observed during the same experiment (Figure S23). The selectivity decrease observed for both Pd and Pd–Au with the increase in acetylene concentration can be explained by enhanced carbon deposition with increasing acetylene partial pressure: as the conversion values are similar at different acetylene concentrations, there is more unreacted reactant present at the surface with higher acetylene concentrations, which will inevitably cause higher carbon deposition. To validate this idea, the selectivity values for higher hydrocarbon products (n‐butane, n‐butene, trans‐butene, cis‐butene, and 1,3‐butadiene) are calculated and presented in Figure S24. For both Pd and Pd–Au LCCs, there is an increase in C_4_ products with the increase in acetylene concentration (more pronounced for Pd–Au than for Pd). It is known that the formation of higher hydrocarbons leads to oligomerization and polyacetylene formation during the acetylene hydrogenation, which leads to blocking of active sites and consequent deactivation of the catalyst [10].

It is evident from this study that the Pd:C phase formed during reaction is a crucial parameter affecting the activity of the semi‐hydrogenation catalyst. For the Pd LCC, the population of the Pd:C species at the surface affects the ethylene production, i.e. higher Pd:C concentration result in higher ethylene yield. By regulating the carbon amount, one can clearly observe changes in the conversion of acetylene, where the control over the carbon content in the Pd lattice is achieved by the addition of Au into the structure, which exhibits less susceptibility for carbon incorporation, as evidenced by the spectroscopic results. The incorporated carbon also appears to influence the activity significantly, and the second metal (in this case Au) modifies the amount of carbon that can be incorporated into the Pd lattice. The addition of Au results in lower solubility of C in the Pd lattice, which causes lower activity for acetylene hydrogenation. Therefore, there is no need to add Au into the Pd lattice to enhance the performance for this reaction ‐ on the contrary, it seems to have an adverse effect. Furthermore, the catalyst transforms under the reaction conditions, as unveiled by the significantly different structures of the Pd and Pd–Au films with the inclusion of carbon in their structure, which influences the activity during the reaction. The as‐grown laterally condensed catalyst in this case acts only as a pre‐catalyst and transforms into the ‘real’, active catalyst throughout different stages of the reaction (sub‐sequential heating steps or increasing reactant concentration applied in this study), gradually incorporating a higher amount of carbon in the metal catalyst lattice. From the comparison of 1 mbar and 900 mbar experiments, it can be concluded that the reaction at these two different pressures causes similar changes in the electronic and the chemical/atomic structure, i.e., Pd:C phase formation and subsequent lattice expansion. That means that there is no pressure gap for this reaction between mbar and bar range and that the catalyst can be investigated by NAP‐XPS at mbar conditions. Thanks to the easier access of spectroscopic tools to the LCC structure, we could characterize the operando surface and sub‐surface transformations of this catalyst, leading to the design of working catalysts with enhanced activity.

## 4 Conclusions

In this study, the electronic structures of thin (3 nm) Pd and Pd–Au layers in the form of laterally condensed catalyst structures were investigated during the selective acetylene hydrogenation reaction by means of operando X‐ray photoelectron spectroscopy and near‐edge absorption fine structure spectroscopy. The NAP‐XPS experiments were performed in the mbar range, where the hydrogen partial pressure is not sufficient to form Pd hydride species, thus enabling the exploration of the influence of the carbon incorporation into the Pd and Pd–Au lattices without having to disentangle effects coming from the hydride phase. From the analysis of the XPS data, we have found that Pd can incorporate significantly more carbon atoms into its structure than Pd–Au, due to a weaker interaction between Au and C atoms. This observation is also validated in XAS measurements performed at both 1 and 900 mbar, i.e. the spectral features related to the Pd:C phase formation and subsequent lattice expansion are observed in Pd and not in Pd–Au, indicating the absence of a pressure gap for this reaction. The results are complemented by DFT calculations, where a gradual addition of Au into the Pd lattice causes the decrease of the maximum carbon content. It can be concluded that there is no need to add Au into the Pd lattice, as its presence causes less C incorporation into the lattice, resulting in lower catalytic activity. Owing to the possibilities of operando experiments, the dynamic transformation of the carbon atoms dissolved in the palladium lattice during the selective acetylene hydrogenation reaction could be observed, which would be overlooked by ex situ analyses. The present study leads the way for a better understanding of the influence of interstitial carbon atoms dissolved in Pd catalysts, their transformation by addition of a second element (Au) in the selective hydrogenation reactions, and how its incorporation can modify the catalytic activity toward selective acetylene hydrogenation reaction. The catalytic activity of Pd can be tuned with the controlled addition of a second element by the modification of the electronic structure.

## 5 Experimental Section

### Sample Preparation

Laterally condensed Pd and Pd–Au catalysts used in this work were synthesized via physical vapor deposition (PVD, sputtering) and SiO_2_ as barrier layer was deposited by plasma‐enhanced chemical vapor deposition (PECVD). Czochralsky grown, six‐inch silicon wafers were used as substrate material. To separate the wafer into individual sample pieces, we employed UV laser ablation (Keyence MD‐U) to structure the backside of the wafers. After structuring, the samples were separated into 5 × 5 cm^2^ plates and wet chemically cleaned afterwards. For cleaning, a conventional RCA (Radio Corporation of America) treatment was performed by submerging the samples in an ammonia solution, followed by aqueous hydrogen peroxide solution. Both steps were performed at 75°C for 7 min. To remove the native oxide formed during the steps, an HF dip (1% HF) and DI water rinse were performed. The final native oxide was removed immediately before coating of the silicon dioxide. Silicon dioxide buffer (20 nm) was deposited by PECVD using 60 MHz excitation at 200 W. Precursor gases were monosilane (SiH_4_) and nitrous oxide (N_2_O) at flow ratio of 1:40. The substrate temperature was kept constant at 400°C. After buffer deposition, the samples were transferred under a nitrogen atmosphere to a UHV sputtering chamber (PREVAC), which was part of the infrastructure of the Energy Materials In situ Laboratory (EMIL) at Helmholtz‐Zentrum Berlin für Materialien und Energie (HZB). Palladium and palladium‐gold layers were deposited by RF magnetron sputtering at room temperature. Nominal 3 nm (with accuracy of ±1 nm) thin films were synthesized using a 3N5 2‐inch palladium (and a 4N gold) target, Argon flow rate of 1.8 sccm, a 13.56 MHz RF plasma operated at 50 W for Pd and 20 W for Au (equivalent to a nominal Pd:Au ratio of 50:50), and a resulting sputter pressure of 4x10^−3^ mbar. The base pressure of the sputter chamber is 1x10^−8^ mbar.

The granules of Pd (EVOCHEM, 99.99%) and Au (EVOCHEM, 99.99%) were used for the synthesis of the bulk Pd‐Au reference samples. These components were weighted in atomic ratios corresponding to the nominal compositions of Pd_3_Au, PdAu, and PdAu_3_. As references, elemental Pd and Au were prepared in an analogous way. The weighted elements were melted in the arc melter with the water‐cooled copper mold under inert argon atmosphere. To achieve the homogeneity, the ingots were melted 3–4 times accompanied by turning the ingots. The mass losses during the arc melting did not exceed 0.2%. The obtained ingots were placed into ZrO_2_ crucibles and sealed into Ta ampoules under protective Ar atmosphere. Afterwards, sealing into quartz tubes was carried out to protect the Ta ampoules from the oxidation upon annealing. The homogenization annealing was performed in the resistance furnace at 1223 K for 30 days, followed by quenching in the iced water with simultaneous break of the quartz ampoules.

### X‐ray Photoelectron Spectroscopy and Near Edge X‐ray Absorption Fine Structure

NAP‐XPS and NAP‐NEXAFS measurements were carried out at the BElChem (UE56/2‐PGM1) [47] and CAT@EMIL [48] (UE48‐PGM and U17‐DCM beamlines) at the BESSY II electron storage ring operated by the Helmholtz‐Zentrum Berlin für Materialien und Energie. The measurements in NAP conditions were performed with a hemispherical analyzer (SPECS PHOIBOS 150 NAP) equipped with a differential pumping stage, details are provided elsewhere [49]. The Pd and Pd–Au samples used in this study consist of the following composition: 3 nm Pd/20 nm SiO_2_/Si (100) and 3 nm Pd–Au/20 nm SiO_2_/Si (100) and will be named hereafter as Pd and Pd–Au LCC, respectively. The thin film samples were transferred to the beamline via a custom‐made vacuum transfer box from an Argon glovebox without exposing them to ambient atmosphere. Experimental details regarding sample mounting and heating are described elsewhere [10]. Ambient pressure (900 mbar) NEXAFS experiments were performed by using a custom‐made ambient pressure cell. A three dimensional drawing of the cell is provided in Figure S1. The cell was closed with a lid that had a 100 nm thick SiN_*x*_ membrane glued on, which was transparent to the X‐rays, making XAS measurements possible but not XPS. For the ambient pressure XAS measurements, the samples were briefly exposed to air during the sample mounting. The samples were first reduced in 30% H_2_ in He at 125°C for 30 min, and then cooled down to room temperature before introducing the reaction mixture.

All the binding energies reported in this study were corrected with respect to the Fermi edge position (inflection point) recorded with the same photon energy used for the measurement of the core level. Pd 3d spectra were deconvoluted by using a custom‐made fitting software written in Python using the non‐linear least squares fitter from the lmfit package [50], allowing for the incorporation of background lineshapes as active fit components that were optimized simultaneously with the peak components. As background correction, Shirley background was used in combination with a polynomial background to compensate the changes in the background due to excess carbon accumulation during the operando acetylene hydrogenation. Pd 3d core levels were deconvoluted using Doniach‐Sunjic lineshape convoluted with a Gaussian for the metallic (or alloy) Pd, Pd:C, and their corresponding plasmon loss peaks. The asymmetry parameter for the Pd metal peak was used from the values determined by Hüfner et al. (α = 0.18) [51], whereas the asymmetry of the Pd:C (Pd incorporated with C) peak is fixed to 0.11. For the Pd–Au samples, a lower asymmetry value is used for the Pd–Au alloy peak and Pd:C peaks compared to the pure Pd samples (α = 0.16 for Pd–Au alloy and α = 0.08 for Pd:C peak). For the spectra acquired under operando acetylene hydrogenation conditions, a set of additional peaks at higher binding energies was observed (348 eV), which originate from the inelastic scattering of photoelectrons by hydrogen molecules and was reported previously for a Pd foil measured in situ in H_2_ atmosphere using NAP‐XPS [52]. Au 4f and C 1s spectra were deconvoluted by using Doniach‐Sunjic lineshape convoluted with a Gaussian with very small asymmetry values. Quantification was performed by normalizing the peak areas to the photon flux, photoionization cross‐section, and orbital asymmetry values [53, 54, 55]. The values for the inelastic mean free path of the electrons (IMFP) are calculated according to the ref. [56]. The fit parameters are provided in Tables S1–S4.

NEXAFS measurements were performed in the total electron yield (TEY) mode. Background normalization of the NEXAFS Pd L_3_‐edge spectra was performed by using the Athena software (Demeter 0.9.26 package) [57]. The NEXAFS spectra measured under mbar conditions were energy corrected with respect to the Fermi edge position of the valence band spectra acquired at a photon energy of 3160 eV. A similar energy calibration could not be conducted for the ambient pressure measurements, as it was not possible to perform XPS measurements with the closed SiNx membrane. Therefore, the same magnitude of energy correction observed in the UHV, RT conditions (fresh sample) during the NAP‐XPS measurements was applied to the spectra acquired with the ambient pressure cell. The lattice constants of the LCC samples were also calculated from the analysis of the NEXAFS Pd L_3_‐edge spectra. First, the peak observed at around 3203 eV in the Pd and Pd‐Au bulk reference samples (Figure S7) was fitted with a single peak having Voigt lineshape and the peak center was located. The linear correlation between the peak center and the lattice constant measured by XRD (Figure S8) was then propagated to calculate the lattice constant of the LCC samples based on their peak position at ca. 3203 eV in the Pd L_3_‐edge spectra. Furthermore, the calculated lattice constants of the LCC samples from the NEXAFS analysis were used to quantify the carbon amount dissolved in the Pd (or Pd–Au lattice) by using the equation a = a_0_ × 0.69x, where a is the calculated lattice constant, a_0_ is the lattice constant of metallic Pd, and x is the stoichiometric factor of carbon in the PdC_*x*_ structure [42].

### Gas Analytics

The catalytic activity of Pd and Pd–Au LCCs towards the selective acetylene hydrogenation reaction was evaluated by gas chromatography (GC) measurements, which was possible to perform during the NAP‐XPS and NAP‐NEXAFS since the GC (Thermo Scientific TRACE 1300) was attached to the setup in a way that the outlet of the XPS cell (or the ambient pressure reaction cell) was connected to the GC inlet, making product detection possible under reaction conditions. Product analysis was performed by use of a Flame Ionization Detector. The total pressure during the NAP‐XPS experiments was 1 mbar with C_2_H_2_ to H_2_ ratio of 1:10. A blank measurement with an empty sample holder was conducted under the same reaction conditions and the conversion was corrected according to the blank noise signal. The conversion (X) and selectivity (S) values were calculated according to the following formulas:

$$X=\left[\frac{C_{2}H_{4}+C_{2}H_{6}+2*C_{4}H_{8}+2*C_{4}H_{10}}{C_{2}H_{2}+C_{2}H_{4}+C_{2}H_{6}+2*C_{4}H_{8}+2*C_{4}H_{10}}*100\right]$$

$$S_{C_{2}H_{4}}=\left[\frac{C_{2}H_{4}}{C_{2}H_{4}+C_{2}H_{6}+2*C_{4}H_{8}+2*C_{4}H_{10}}*100\right]$$

### Electron Microscopy

Scanning transmission electron microscopy and energy‐dispersive X‐ray spectroscopy (STEM‐EDXS) were performed on a Thermo Fisher Scientific Talos F200X equipped with a high brightness field emission gun (XFEG) and a SuperX 4 SDD EDX detector. The microscope was operated at 200 kV. All data were recorded and analyzed using the Velox software (Thermo Fisher). TEM lamella sample cross‐sections were prepared by focussed ion‐beam milling using a TFS Helios NanoLab G3 FIB‐SEM Dualbeam system.

Scanning Electron Microscopy was performed with Hitachi S‐4800. The measurements were performed at an accelarating voltage of 1.5 kV and 3 mm working distance. A low accelerating voltage was used to limit the size of the excitation volume, which, combined with a short working distance, facilitated high‐resolution microscopy.

### X‐ray Diffraction

X‐ray diffraction of the bulk reference samples were performed in Bragg‐Brentano geometry on a Bruker D8 Advance A25 theta/theta diffractometer with CuKα1+2 radiation, equipped with a LYNXEYE XT‐T position sensitive detector in high energy resolution mode (no Ni filter required). The samples were mounted inside a 0.5 mm deep cavity of a silicon single crystal low background sample holder and measured while the sample was spinning. The diffraction patterns were analyzed by whole pattern fitting using DIFFRAC.SUITE TOPAS (v5, 1999‐2014 Bruker AXS).

### Inductively Coupled Plasma Optical Emission Spectrometry (ICP‐OES)

ICP‐OES was utilized to determine the actual composition of the Pd‐Au LCC sample. The sample was exposed to a medium under ambient conditions for 22 h, where the medium consists of 4.0 mL of 36% HCl and 1.32 mL of 65% HNO_3_, adjusted to a total volume of 20 mL with water, excluding the volume of the wafer pieces. Aliquots of 2.0 mL from each solution were diluted to 5 mL with water and subsequently analyzed using ICP‐OES. The sample area was determined optically, assessing the shadow in a light array to ensure consistent measurement conditions across all samples.

### DFT Calculations

All DFT calculations were performed with the Quantum Espresso [58, 59] (version 6.6) software package and the revised Perdew‐Burke‐Ernzerhof (RPBE) exchange‐correlation functional [60]. A plane wave basis set describing electronic states with an energy cutoff value of 680 eV was used for all calculations, while applying a Fermi‐Dirac smearing of 0.05 eV. Carbon intercalation in bulk Pd and Pd–Au was modeled in 3×3×3 fcc unit cells. The initial Pd and Au structures were based on bulk‐optimized lattice constants of 3.97 and 4.18 Å, respectively, which were obtained with a 20×20×20 k‐point Monkhorst‐Pack mesh grid [61]. Subsequently, an 8×8×8 k‐point grid was used for all simulated 3×3×3 Pd, Au, and Pd–Au structures. The Pd–Au structures were specifically constructed with a fixed Pd:Au ratio and the unit cell was first optimized without any intercalated carbon. Next, a database of metal‐carbon structures was generated sequentially for an increasing number of carbon atoms within octahedral lattice sites in a similar manner to Ref. [19]. For each carbon concentration, the most energetically favorable configuration was chosen and subjected again to a cell and geometry optimization to obtain the optimal unit cell and total (electronic) energy. All optimizations were relaxed to convergence criteria ⩽0.01 eV/Å for atomic forces and ⩽0.001 eV for the total energy using the Broyden–Fletcher–Goldfarb–Shanno (BFGS) algorithm. Finally, the formation free energies for each carbon‐containing structure were predicted using as reference the free energy of a carbon atom within gaseous phase C_2_H_2_ at standard conditions. Free energy contributions, as well as structural parameters of all optimized simulation cells are listed in Tables S5 and S7–S10.

### Simulated Catalytic Testing in Reactor to Complement Operando Catalytic Data

Acetylene hydrogenation reaction was carried out in a plug‐flow fixed‐bed reactor connected to Agilent GC 7890 equipped with a FID for online hydrocarbons analysis (acetylene, ethylene, ethane, and butane). The inner diameter of the reactor tube was 7 mm. A thin film Pd or Pd–Au catalyst (3x20 mm^2^) was positioned vertically in the reactor, where quartz wool was placed on the top and bottom of the catalyst. The as‐synthesized catalysts were first pretreated in 30% H_2_ (N_2_ balance, 30 mL min^−1^) by heating the reactor from room temperature to 125°C at 5°C min^−1^ and holding at 125°C for 30 min. Afterwards, the temperature was decreased to room temperature under N_2_ atmosphere. For catalytic measurements, a gas mixture of C_2_H_2_ and H_2_ balanced with N_2_ was fed to the reactor for acetylene hydrogenation from room temperature to 125°C, with each temperature step maintained for 2 h. The C_2_H_2_:H_2_ ratio was 1:10 and the partial pressures of C_2_H_2_ and H_2_ were set to be identical to those used in the operando measurements for comparison.

## Conflicts of Interest

The authors declare no conflicts of interest.

## Supporting information




**Supporting File**: adma71933‐sup‐0001‐SuppMat.pdf.

## Data Availability

The data that support the findings of this study are available from the corresponding author upon reasonable request.
