# Supplementary material for: Operando X‐ray Spectroscopy Study of Pd and Pd–Au Laterally Condensed Catalysts during Selective Acetylene Hydrogenation: The Role of Carbon
Source: Adv Mater. 2026 Jan 10;38(11):e17227. doi: 10.1002/adma.202517227 (PMC12921353; doi:10.1002/adma.202517227)
Supplement: Supplementary file 1 — Supporting File: adma71933‐sup‐0001‐SuppMat.pdf. [file ADMA-38-e17227-s001.pdf]

Supporting Information

Operando X-ray Spectroscopy Study of Pd and Pd-Au Laterally Condensed Catalysts during Selective Acetylene Hydrogenation: The Role of Carbon

Eylül Öztuna, Thomas Götsch, Daniel Cruz, Patrick Zeller, Olga V. Vinogradova, Zehua Li, Christian Rohner, Franz-Philipp Schmidt, Zahra Gheisari, Alexander Steigert, Martin Muske, Michael Hävecker, Eugen Stotz, Iryna Antonyshyn, Frank Girgsdies, Olaf Timpe, Thomas Lunkenbein, Vanessa J. Bukas, Karsten Reuter, Robert Schlögl, Katarzyna Skorupska,* Axel Knop-Gericke,* and Beatriz Roldán Cuenya*

E-mail: skorupska@fhi-berlin.mpg.de; knop@fhi-berlin.mpg.de; roldan@fhi-berlin.mpg.de

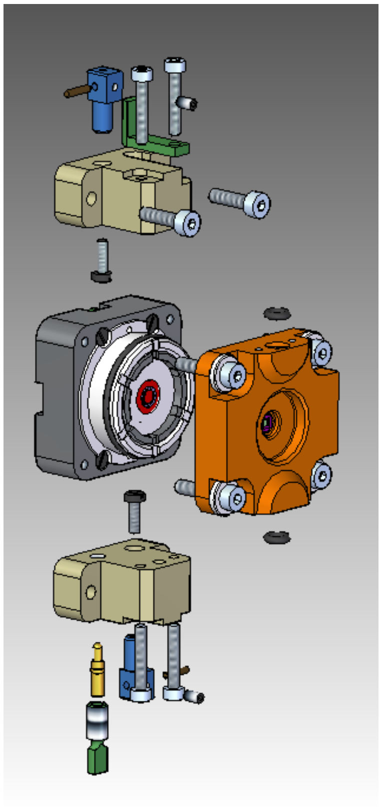

Figure S1: 3D drawing of the ambient pressure cell used in this study. Image courtesy: Eugen Stotz from FHI Berlin.

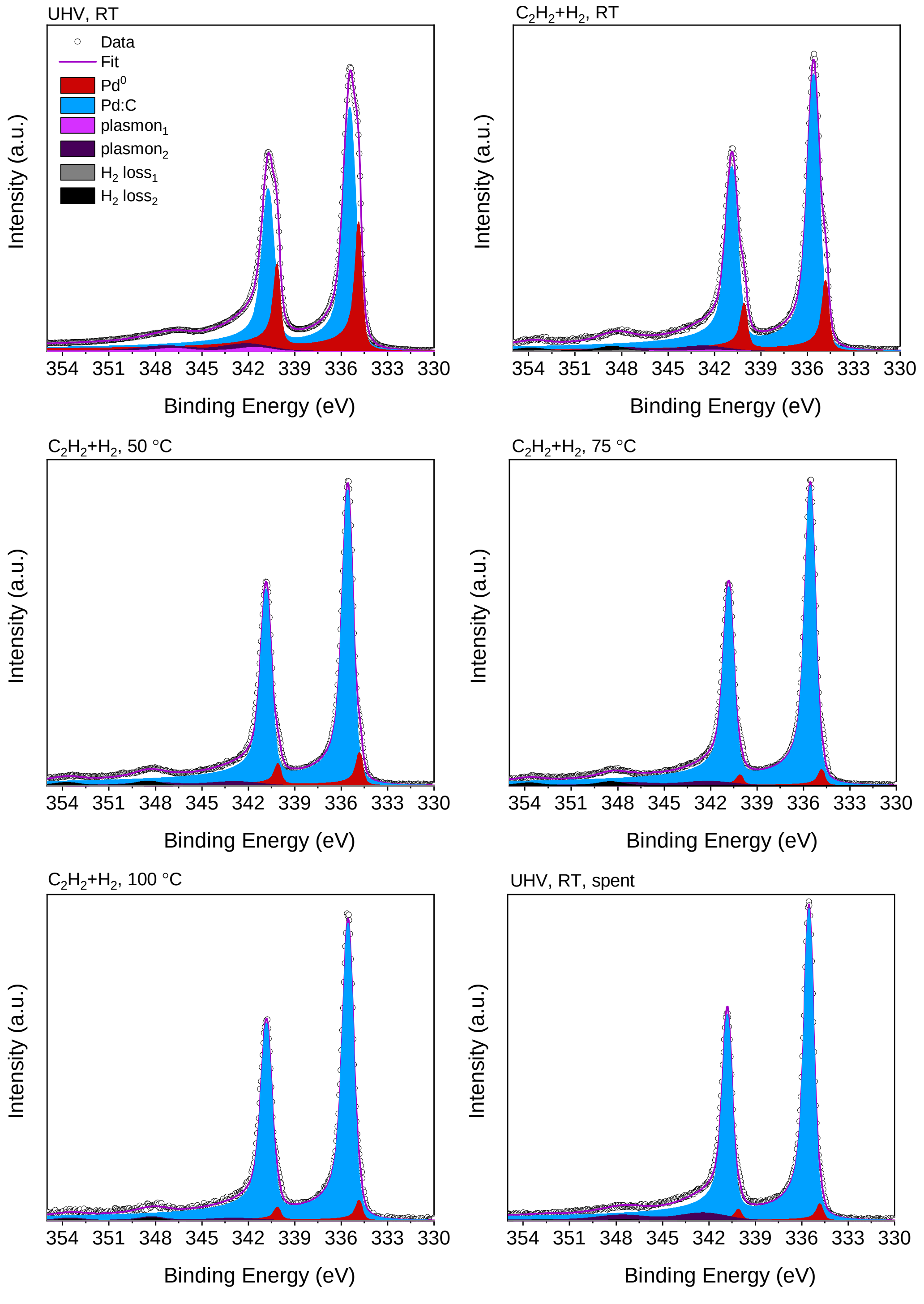

Figure S2: Deconvoluted XPS Pd 3d spectra of Pd LCC. The kinetic energy of photoelectrons is 400 eV, which corresponds to an IMFP of 7 Å. H_2 loss peaks originate from the scattering of photoelectrons through H_2 molecules.

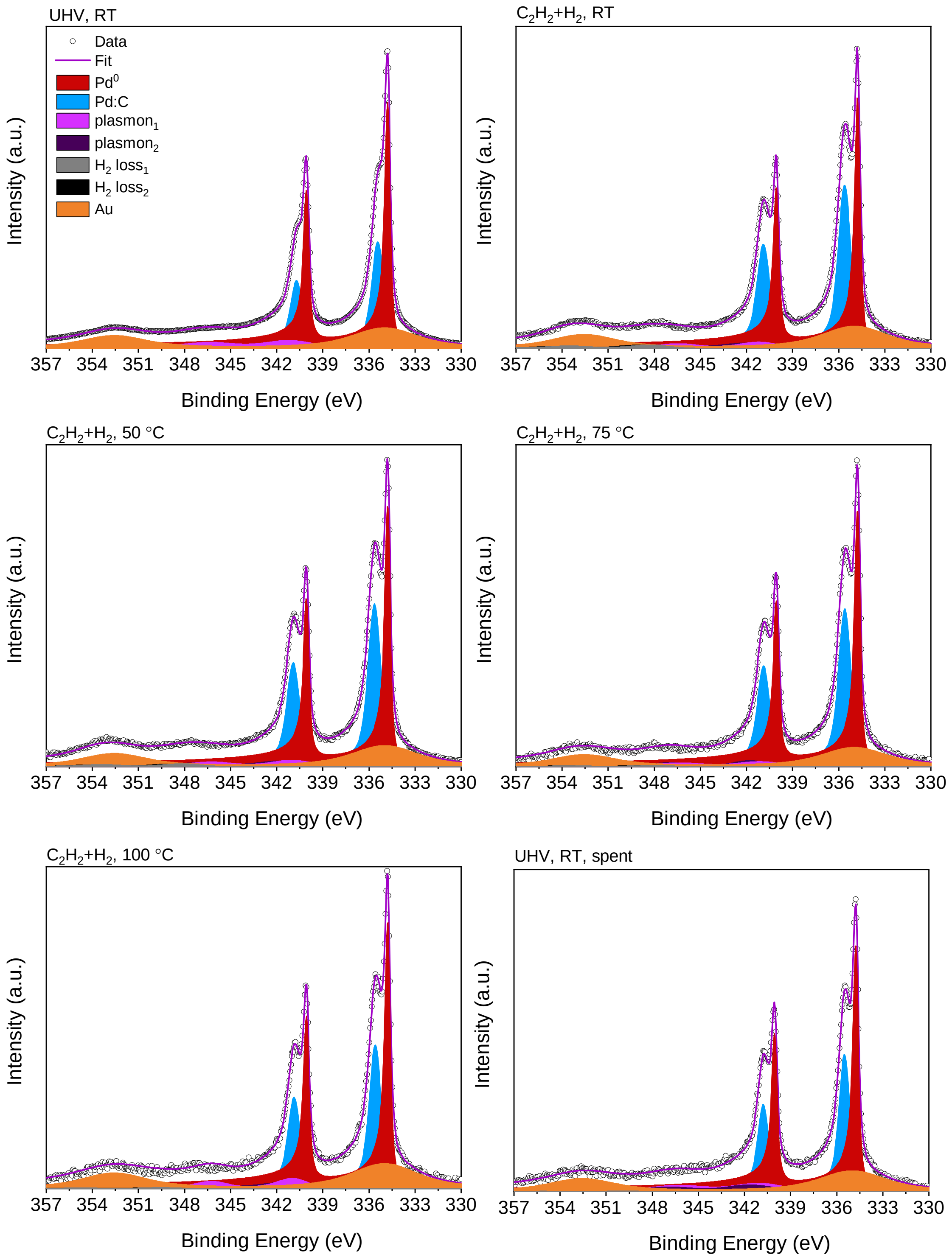

Figure S3: Deconvoluted XPS Pd 3d/Au 4d spectra of Pd-Au LCC. The kinetic energy of the photoelectrons is 400 eV, which corresponds to an IMFP of 7 Å. H_2 loss peaks originate from the scattering of photoelectrons through H_2 molecules.

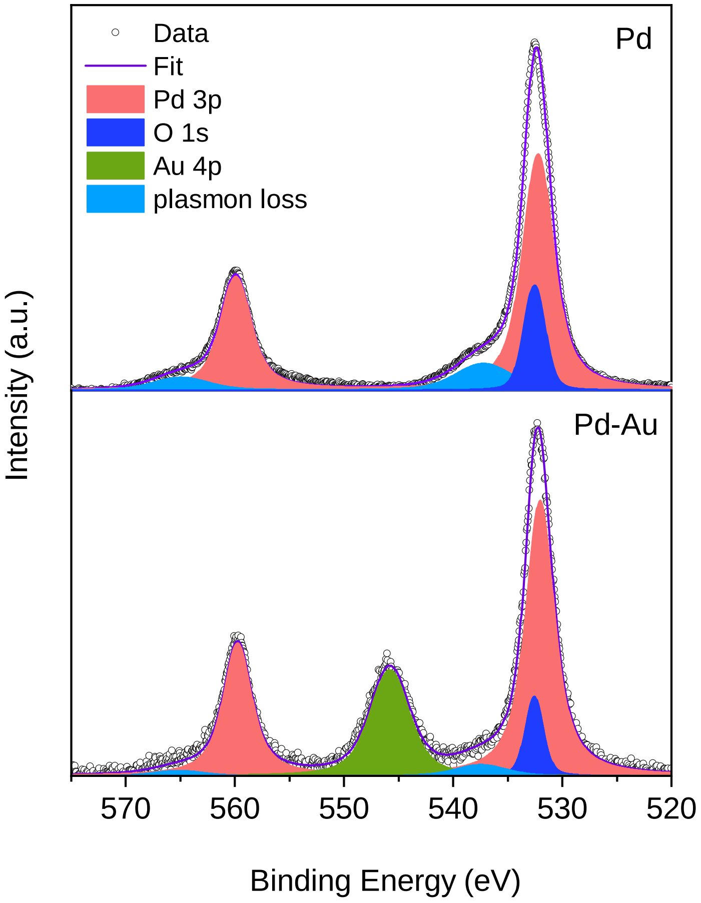

Figure S4: Pd 3p/O 1s spectra of as-prepared Pd and Pd-Au LCCs in UHV and RT. Pink, green, blue, and navy colored curves correspond to Pd 3p, Au 4p, plasmon loss, and O1s of SiO_2 peaks, respectively. The kinetic energy of the photoelectrons is 400 eV, which corresponds to an IMFP of 7 Å.

The Pd $3p_{3/2}$-O 1s spectrum of the Pd LCC is deconvoluted into two peaks at 532.2 eV and 532.5 eV, where the former belongs to Pd 3p of metallic Pd and the latter to O 1s of SiO_2 (Figure S4).[1,2] Surface Pd oxide and bulk Pd oxide peaks were reported at lower binding energies of 529-530 eV.[3,4] Thus, the absence of these peaks in the Pd LCC proves that the sample does not contain any oxidic components. A reference experiment was performed by in-situ heating a Pd foil and a thicker Pd LCC (10 nm) in O_2 environment to determine the surface and bulk Pd oxide spectroscopic features in Pd 3p core level and has been reported in our previous study.[5]

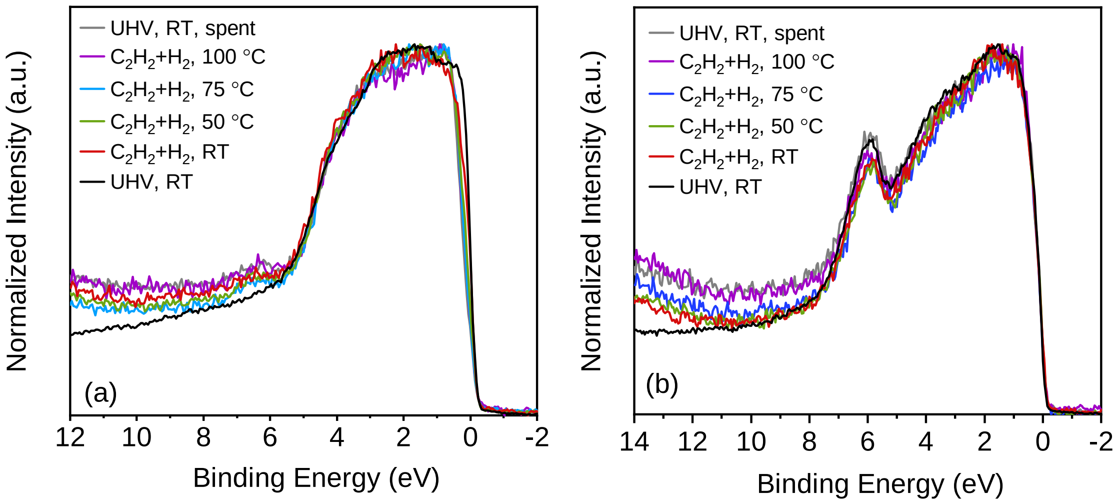

Figure S5: XPS valence band spectra of (a) Pd and (b) Pd-Au LCCs during acetylene hydrogenation. The kinetic energy of the photoelectrons is 700 eV, which corresponds to an IMFP of 10 Å. The total pressure of the experiment is 1 mbar.

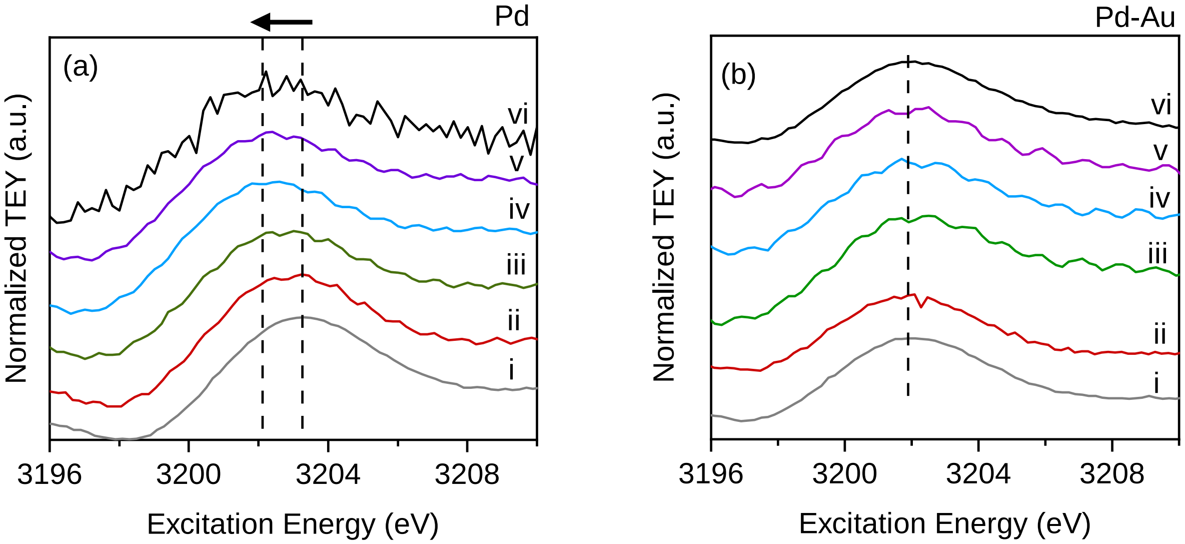

Figure S6: Normalized NEXAFS Pd L_3-edge spectra of (a) Pd LCC and (b) Pd-Au LCC in the energy region of 3196-3210 eV. The measurement conditions are as follows: (i) UHV, RT, fresh (ii) rxn, RT (iii) rxn, 50 °C (iv) rxn, 75 °C (v) rxn, 100 °C (vi) UHV, RT, spent. The vertical dashed lines are guide to the eye to visualize the magnitude of the shift. The total pressure of the experiment is 1 mbar.

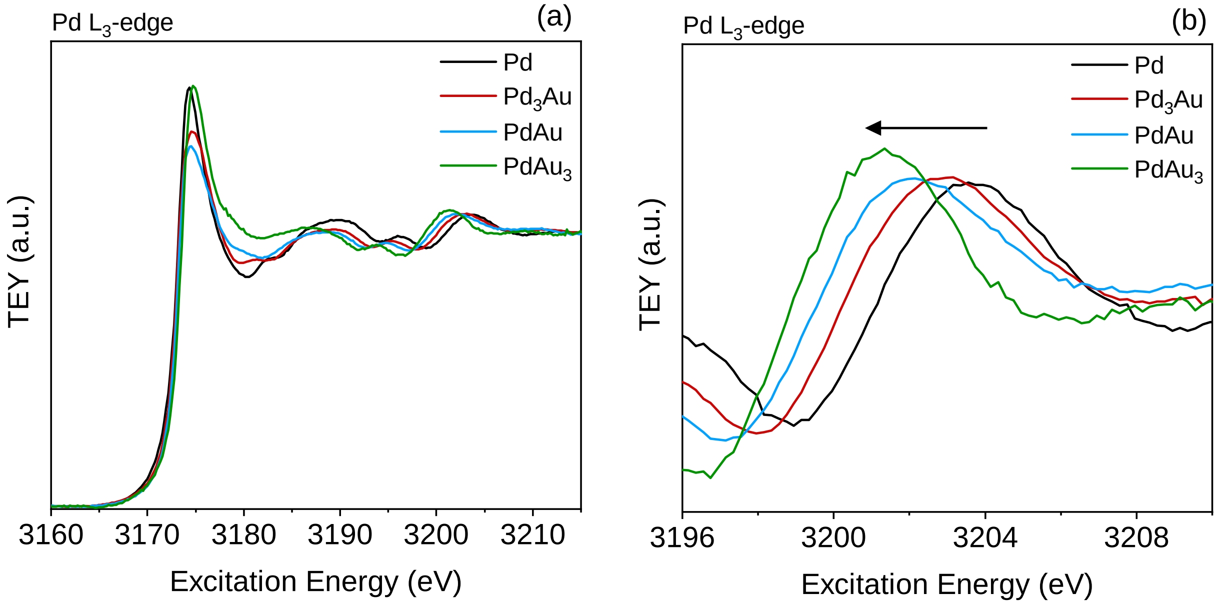

Figure S7: (a) Normalized NEXAFS Pd L_3-edge spectra of Pd and Pd-Au bulk reference samples in different Pd:Au ratios, measured in UHV and RT. (b) shows the enlarged spectra in the energy region 3196-3210 eV.

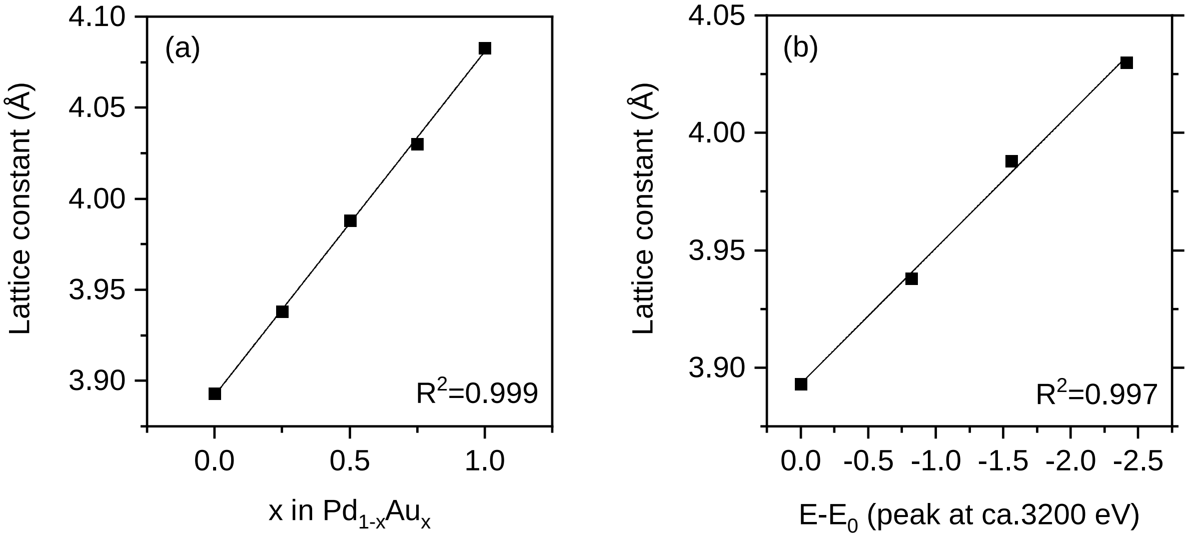

Figure S8: Lattice constants (in Å) measured by XRD (a) vs Au amount in Pd-Au bulk reference samples and (b) vs the energy shift in the peak at ca. 3203 eV in the normalized NEXAFS Pd L_3-edge spectra. E_0 is the energy of the peak at ca. 3203 eV of the pure Pd bulk reference sample.

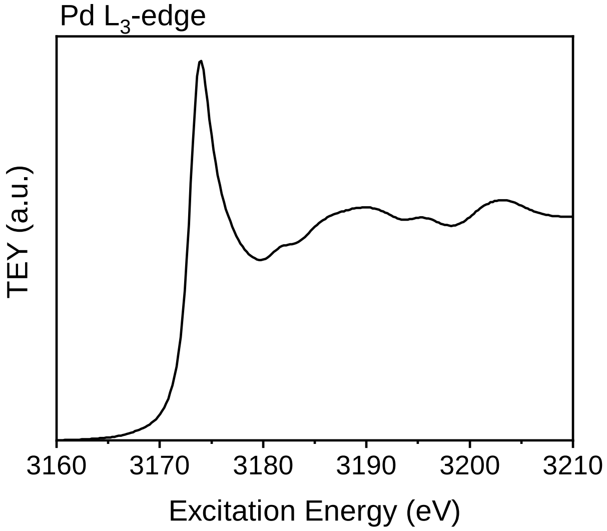

Figure S9: Normalized NEXAFS Pd L_3-edge spectrum of a Pd foil measured at 600 °C in 0.1 mbar O_2.

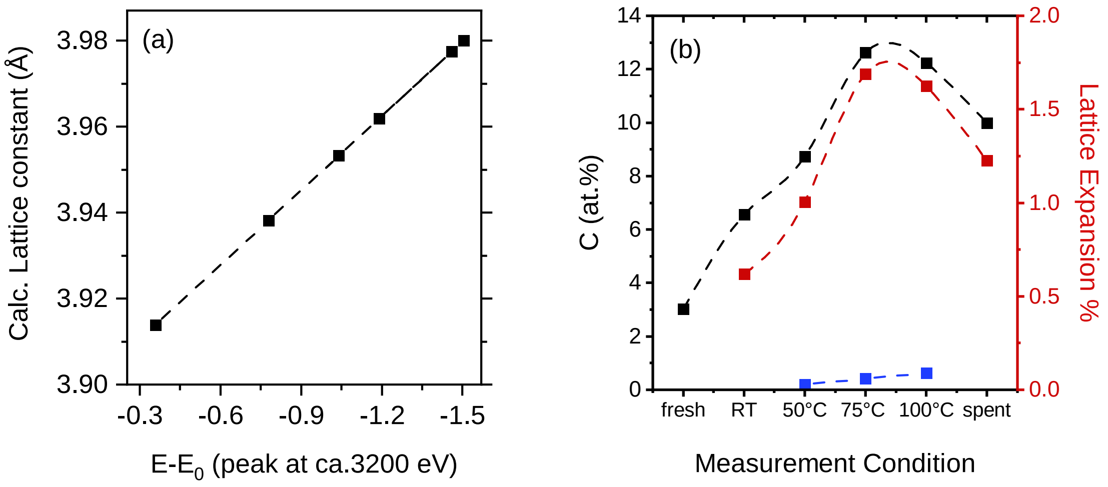

Figure S10: (a) Calculated lattice constants (in Å) of the Pd LCC from the NEXAFS Pd L_3-edge analysis vs the peak shift observed at around 3203 eV. E_0 is the energy of the peak at ca. 3203 eV of the Pd LCC measured in UHV, RT conditions in the as-prepared state. (b) Carbon amount (in at.%) and lattice expansion (in %) calculated from the NEXAFS Pd L_3-edge analysis of the Pd LCC at various stages of the operando 1 mbar acetylene hydrogenation experiment. Blue data points show the amount of thermal lattice expansion at the corresponding reaction temperature. The total pressure of the experiment is 1 mbar.

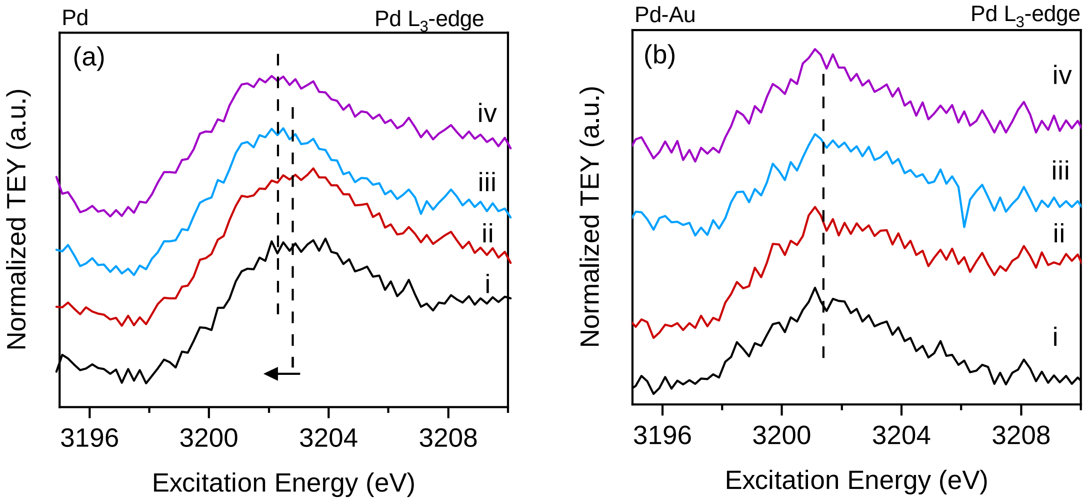

Figure S11: Normalized NEXAFS Pd L_3-edge spectra of (a) Pd LCC and (b) Pd-Au LCC in the energy region of 3196-3210 eV. The measurement conditions are as follows: (i) He (ii) 0.5 % C_2H_2, 100 °C (iii) 1.0 % C_2H_2, 100 °C (iv) 2.0 % C_2H_2, 100 °C. The total pressure of the experiment is 900 mbar. The vertical dashed lines are guide to the eye to visualize the magnitude of the shift.

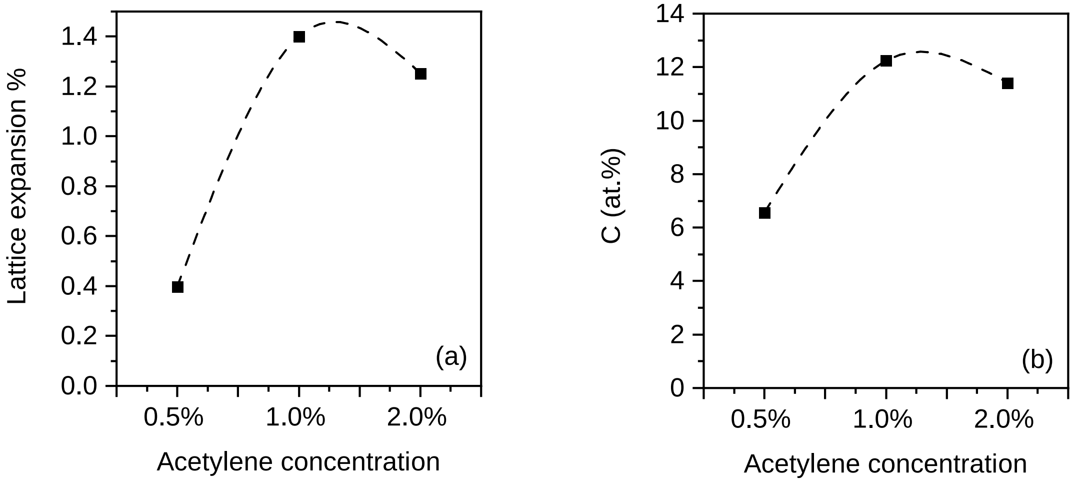

Figure S12: (a) Lattice expansion (in %) and (b) carbon amount (in at.%) calculated from the NEXAFS Pd L_3-edge analysis of the Pd LCC at various stages of the operando acetylene hydrogenation experiment. The total pressure of the experiment is 900 mbar.

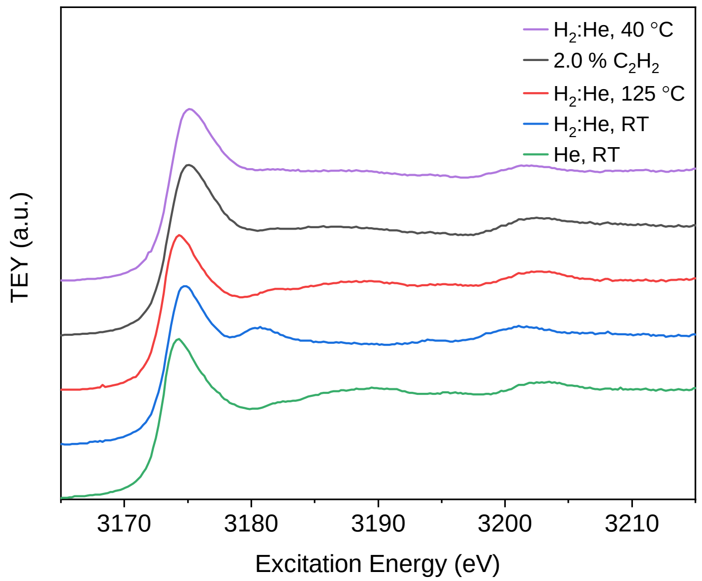

Figure S13: NEXAFS Pd L_3-edge spectra of the Pd LCC. Temperature is 25 °C in the spectrum labelled 'He' and 100 °C during the reaction, which is labelled '2.0 % C_2H_2'. The total pressure of the experiment is 900 mbar.

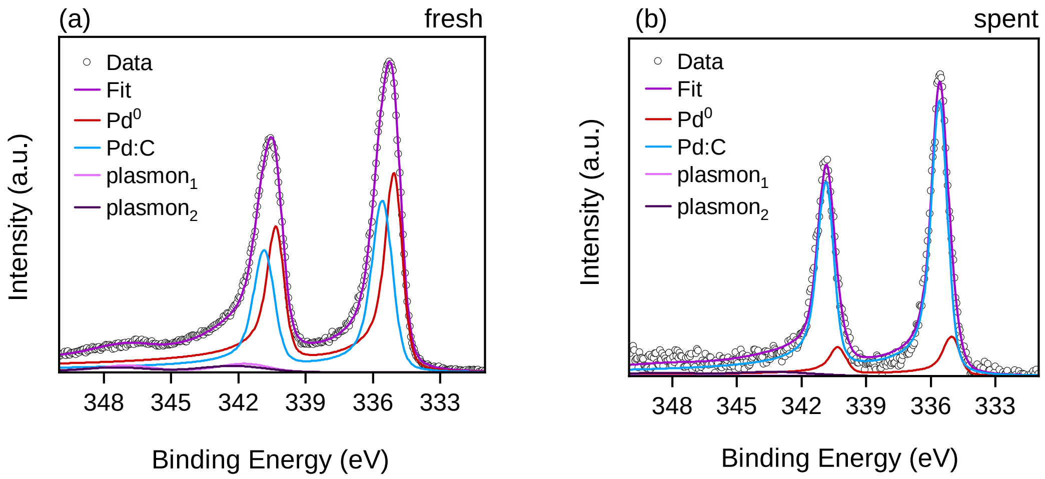

Figure S14: XPS Pd 3d spectra of (a) fresh and (b) spent Pd LCC. Spent sample is produced after acetylene hydrogenation performed at 900 mbar and 100 °C. The photon energy of the measurement is 5000 eV.

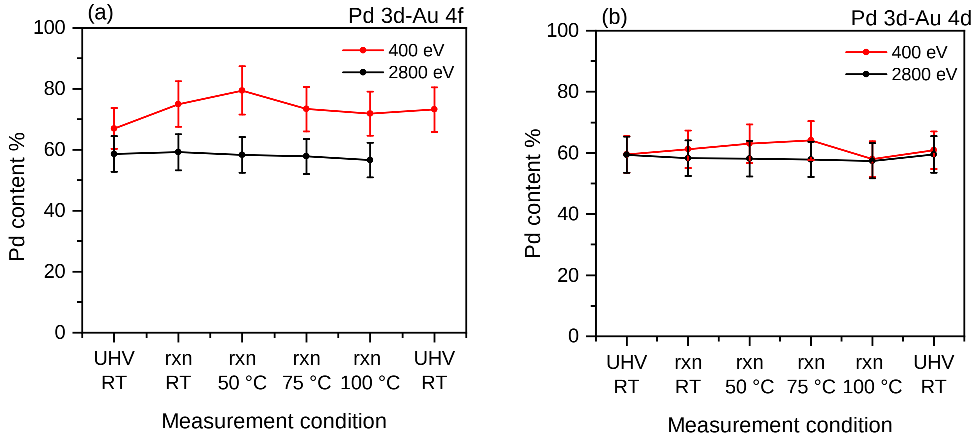

Figure S15: Pd content % (at.) of the Pd-Au LCC calculated from the quantification of (a) Pd 3d-Au 4f and (b) Pd 3d-Au 4d core level spectra during acetylene hydrogenation. The error bars are calculated according to the error margins in the photoionization cross sections.

For the Pd:Au quantification shown in Figure S15, we have selected to use an approximate value of 10% as the uncertainty in the photoionization cross sections. There is a large range of reported values for the cross section or sensitivity factor uncertainty in the literature, i.e. ranging from 5 to 40 %.[6–8] While we are aware that the errors might be higher according to Wagner et al.,[8] the used cross section values anyways affect the absolute numbers in atomic ratio and not the trend between different measurements.

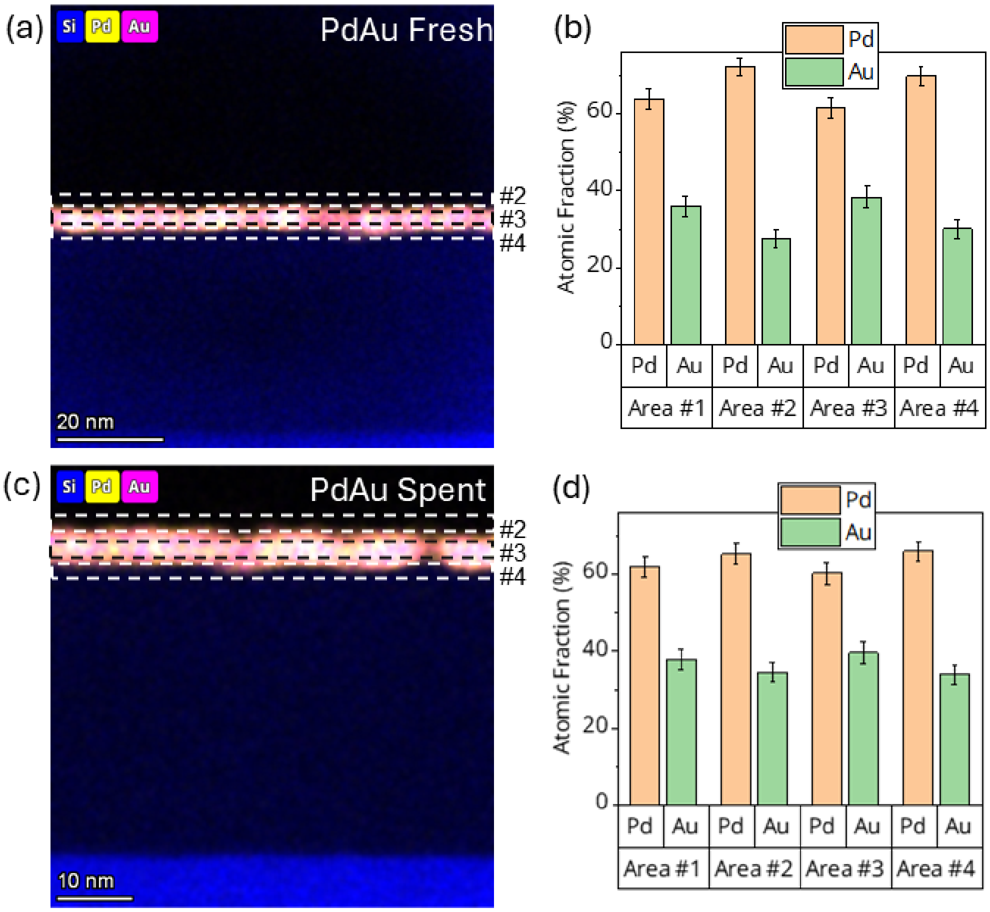

Figure S16: STEM-EDS elemental maps of Pd-Au LCC. (a) EDS map of the fresh as-prepared sample. (b) EDS quantification calculated from integrated spectra of the areas #1-4 indicated by the dashed line boxes. Area #1 corresponds to the full image in (a). (c) EDS map of the sample after operando XPS at 1 mbar (acetylene+hydrogen, temperature increased from RT to 100 °C). (d) EDS quantification from areas #1-4, where area #1 corresponds to the full image in (c).The top surface of the LCC and the interface with the SiO_2-buffer layer have a slightly increased Pd content compared to the body of the metal layer in both, the fresh and the post-operando sample after 1 mbar NAP-XPS experiments. The differences between the two are within the error range.

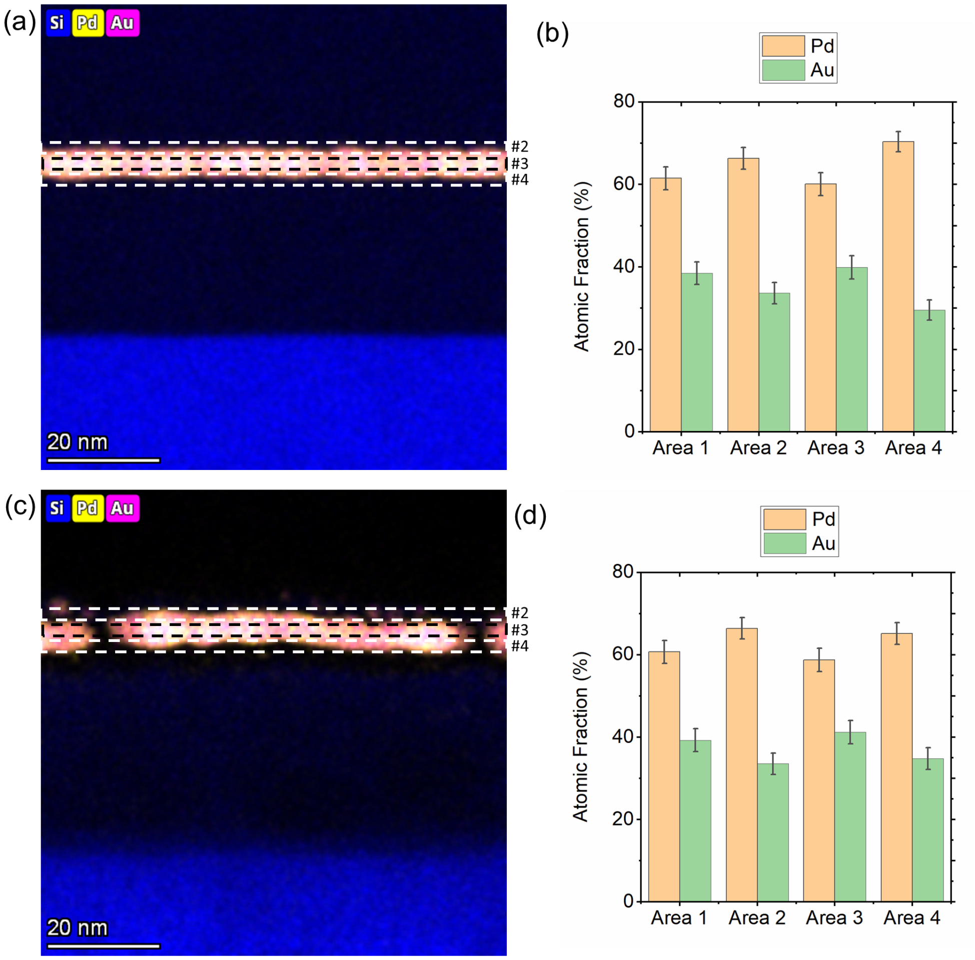

Figure S17: STEM-EDS elemental maps of Pd-Au LCC. (a) EDS map of the fresh as-prepared sample. (b) EDS quantification calculated from integrated spectra of the areas #1-4 indicated by the dashed line boxes. Area #1 corresponds to the full image in (a). (c) EDS map of the sample after operando XAS at 900 mbar (acetylene+hydrogen, where acetylene concentration increased from 0.5 to 2.0 % at a temperature of 100 °C). (d) EDS quantification from areas #1-4, where area #1 corresponds to the full image in (c).

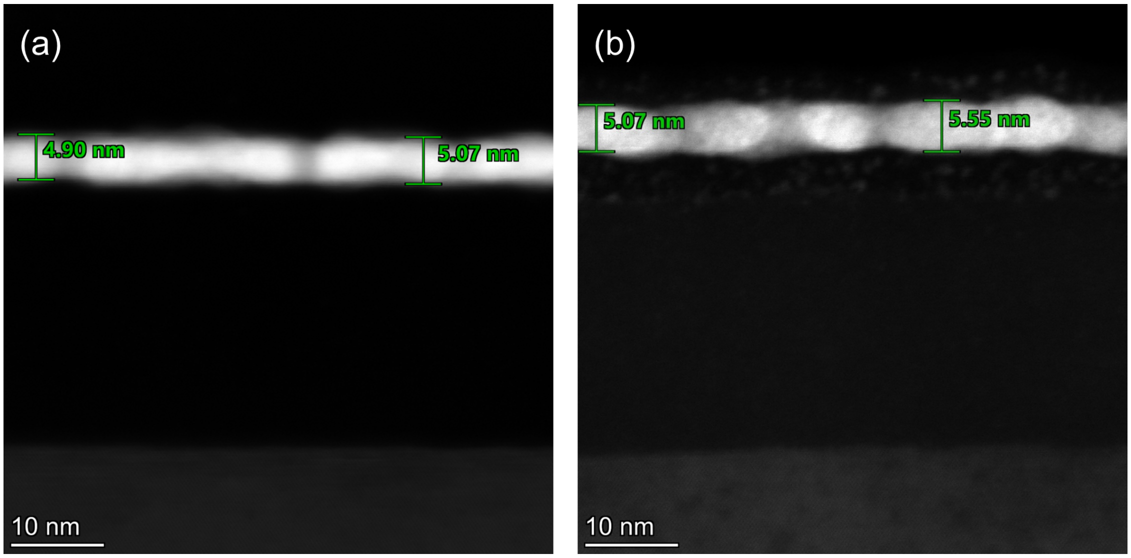

Figure S18: STEM cross-section micrographs of (a) as-prepared Pd-Au LCC and (b) after the operando XAS measurements performed at 900 mbar (acetylene+hydrogen).

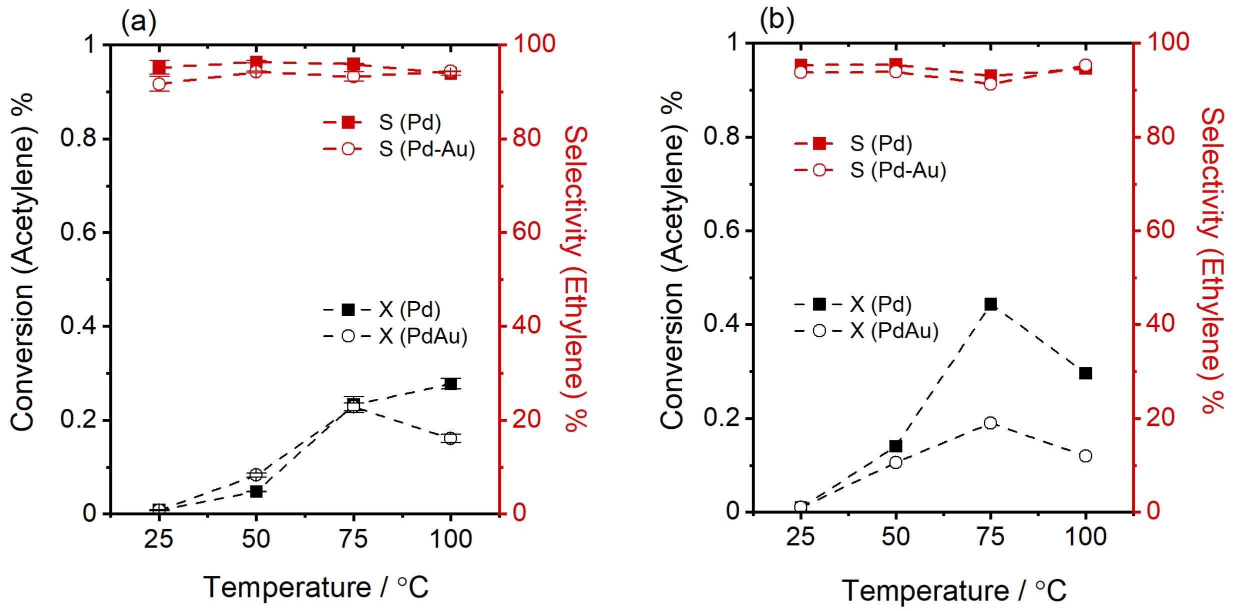

Figure S19: GC results of Pd and Pd-Au LCCs at 1 mbar and various reaction temperatures. (a) includes repetition of measurements performed in the same beamline setup without the beam and (b) includes operando measurements. All the measurements are performed at the NAP-XPS chamber.

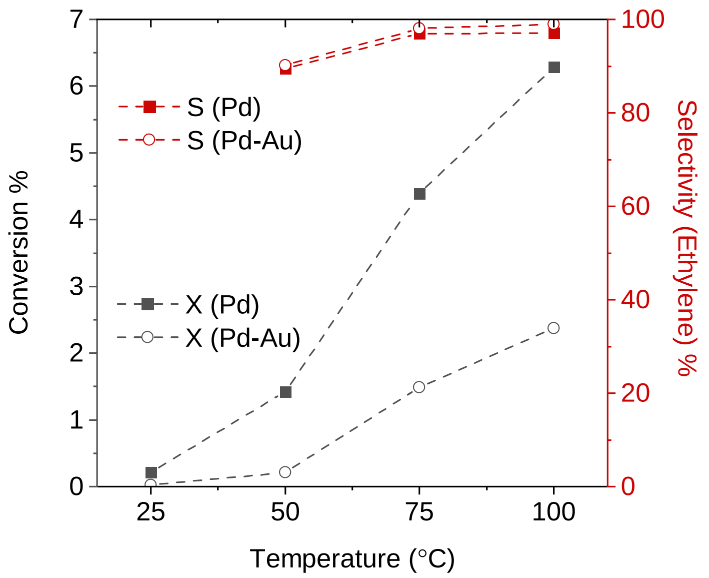

Figure S20: GC results of Pd and Pd-Au LCCs at 1 mbar and various reaction temperatures. The measurements are performed at a fixed bed reactor.

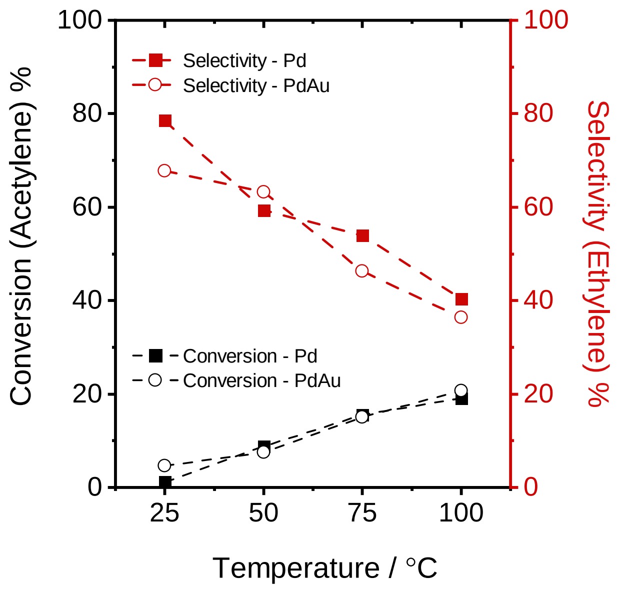

Figure S21: GC results of Pd and Pd-Au LCCs at 900 mbar and various reaction temperatures. The measurements are performed using an ambient pressure reaction cell.

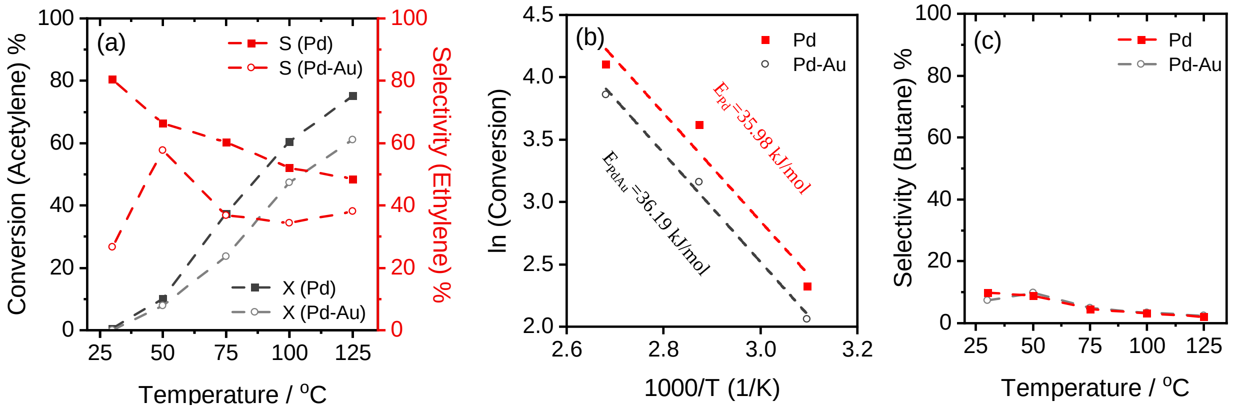

Figure S22: (a) Catalytic activity and ethylene selectivity, and (b) Arrhenius plots for both LCCs where the dashed lines represent the fitted results. The slope of each was used to calculate the activation energy, with the corresponding values shown alongside the plots.(c) Butane selectivity of Pd and Pd-Au LCCs in acetylene hydrogenation from 30 to 125 °C. The C_2H_2 concentration is 2.0% with an C_2H_2:H_2 ratio of 1:10. The total flow is 12.5 mL/min at 1 bar.

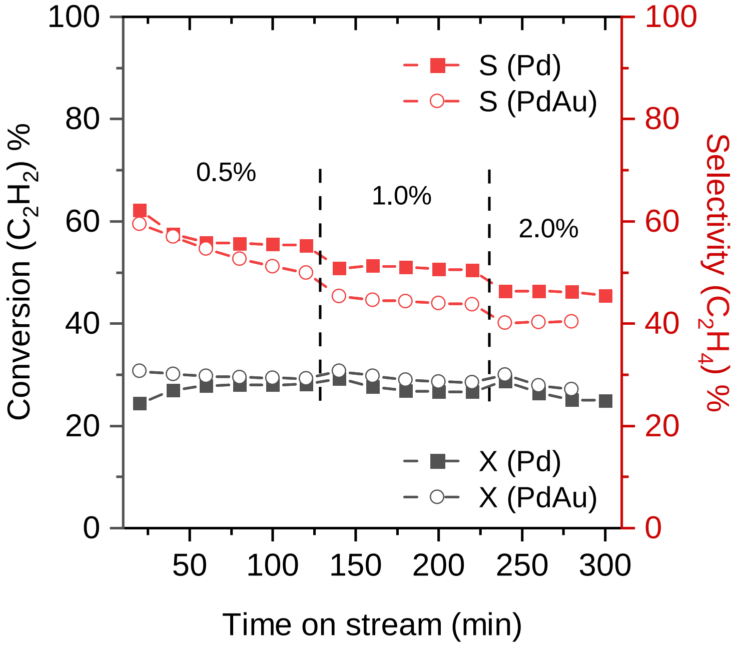

Figure S23: Activity of Pd and Pd-Au LCCs measured time on stream during the acetylene hydrogenation performed at 900 mbar using the ambient pressure reaction cell. C_2H_2:H_2 ratio is 1:10 and C_2H_2 concentrations are indicated on the plot as 0.5, 1.0, and 2.0%. He is used as a balance gas. The temperature is 100 °C.

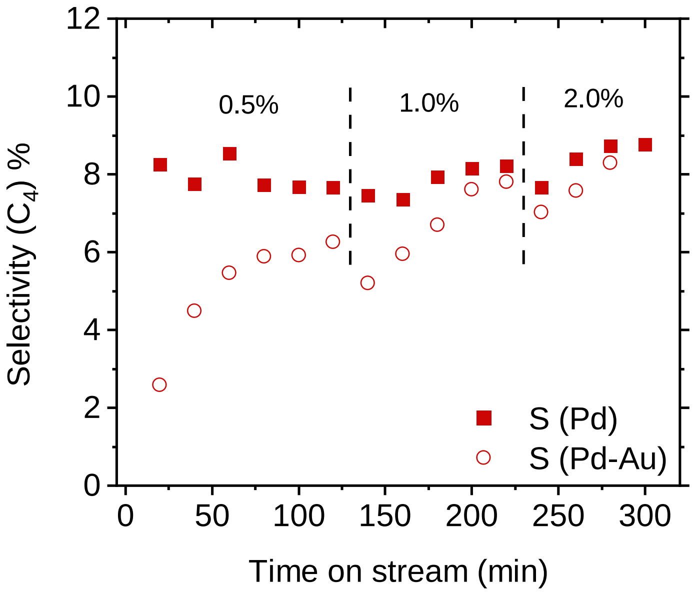

Figure S24: Selectivity to C_4 products (n-butane, n-butene, trans-butene, cis-butene, and 1,3-butadiene) measured time on stream during the acetylene hydrogenation performed at 900 mbar using the ambient pressure reaction cell. C_2H_2:H_2 ratio is 1:10 and C_2H_2 concentrations are indicated on the plot as 0.5, 1.0, and 2.0%. He is used as a balance gas. The temperature is 100 °C.

Table S1: XPS fit parameters for Pd 3d spectra of the Pd LCC during the operando acetylene hydrogenation. C_2H_2:H_2=1:10 and the total pressure is 1 mbar. The kinetic energy of the photoelectrons is 400 eV, which corresponds to an IMFP of 7 Å.

**Condition**	**Parameter**	**Pd(0)**	**Pd:C**
RT in UHV	Binding energy (eV)	334.9	335.4
	FWHM (eV)	0.47	0.56
RT in C_2H_2+H_2	Binding energy (eV)	334.8	335.6
	FWHM (eV)	0.47	0.76
50 °C in C_2H_2+H_2	Binding energy (eV)	334.8	335.6
	FWHM (eV)	0.47	0.76
75 °C in C_2H_2+H_2	Binding energy (eV)	334.8	335.5
	FWHM (eV)	0.47	0.73
100 °C in C_2H_2+H_2	Binding energy (eV)	334.8	335.5
	FWHM (eV)	0.47	0.71
RT in UHV, after rxn	Binding energy (eV)	334.8	335.5
	FWHM (eV)	0.47	0.71

Table S2: XPS fit parameters for C 1s spectra of the Pd LCC during the operando acetylene hydrogenation. C_2H_2:H_2=1:10 and the total pressure is 1 mbar. The asymmetry parameter of all peaks is 0.005 except sp^2-C, which is fitted with asymmetry of 0.095. The kinetic energy of photoelectrons is 400 eV, which corresponds to an IMFP of 7 Å.

Condition	Parameter	Pd:C	sp^2	disordered C	C-H	C-O	C=O
RT in UHV	BE (eV)	284.1	284.3	284.8	285.3	286.0	288.0
	FWHM (eV)	0.55	0.85	0.79	0.99	0.99	0.99
RT in C_2H_2+H_2	BE (eV)	284.1	284.3	284.8	285.3		
	FWHM (eV)	0.55	0.85	0.79	0.99		
50 °C in C_2H_2+H_2	BE (eV)	284.1	284.3	284.7	285.3		
	FWHM (eV)	0.53	0.44	0.9	0.64		
75 °C in C_2H_2+H_2	BE (eV)	284.1	284.3	284.7	285.3		
	FWHM (eV)	0.57	0.43	0.96	0.66		
100 °C in C_2H_2+H_2	BE (eV)	284.1	284.3	284.8	285.3		
	FWHM (eV)	0.43	0.47	0.92	0.77		
RT in UHV	BE (eV)	284.1	284.3	284.8	285.3		
after rxn	FWHM (eV)	0.43	0.52	0.9	0.99		

Table S3: XPS fit parameters for Pd 3d spectra of the Pd-Au LCC during the operando acetylene hydrogenation. C_2H_2:H_2=1:10 and the total pressure is 1 mbar. The kinetic energy of photoelectrons is 400 eV, which corresponds to an IMFP of 7 Å.

**Condition**	**Parameter**	**Pd-Au**	**Pd:C**
RT in UHV	Binding energy (eV)	334.8	335.4
	FWHM (eV)	0.40	0.88
RT in C_2H_2+H_2	Binding energy (eV)	334.8	335.6
	FWHM (eV)	0.40	0.95
50 °C in C_2H_2+H_2	Binding energy (eV)	334.8	335.6
	FWHM (eV)	0.40	0.95
75 °C in C_2H_2+H_2	Binding energy (eV)	334.8	335.6
	FWHM (eV)	0.40	0.93
100 °C in C_2H_2+H_2	Binding energy (eV)	334.8	335.6
	FWHM (eV)	0.40	0.89
RT in UHV, after rxn	Binding energy (eV)	334.8	335.5
	FWHM (eV)	0.40	0.81

Table S4: XPS fit parameters for C 1s spectra of the Pd-Au LCC during the in-situ acetylene hydrogenation. C_2H_2:H_2=1:10 and the total pressure is 1 mbar. The asymmetry parameter of all peaks is 0.005 except sp^2-C, which is fitted with asymmetry of 0.095. The kinetic energy of photoelectrons is 400 eV, which corresponds to an IMFP of 7 Å.

Condition	Parameter	Pd:C	sp^2	disordered C	C-H	C-O	C=O
RT in UHV	BE (eV)	284.1	284.4	284.8	285.3	286.0	287.7
	FWHM (eV)	0.72	0.97	0.99	0.85	0.67	0.99
RT in C_2H_2+H_2	BE (eV)	284.0	284.4	284.8	285.3	286.2	287.9
	FWHM (eV)	0.52	0.51	0.98	0.99	0.98	0.95
50 °C in C_2H_2+H_2	BE (eV)	284.0	284.4	284.8	285.3		
	FWHM (eV)	0.51	0.56	0.74	0.68		
75 °C in C_2H_2+H_2	BE (eV)	284.0	284.4	284.8	285.3		
	FWHM (eV)	0.57	0.54	0.71	0.66		
100 °C in C_2H_2+H_2	BE (eV)	284.1	284.4	284.8	285.3		
	FWHM (eV)	0.51	0.51	0.89	0.86		
RT in UHV	BE (eV)	284.0	284.4	284.8	285.3		
after rxn	FWHM (eV)	0.51	0.83	0.97	0.99		

Table S5: DFT computed zero point energy (ZPE) and entropic contributions (TS) used in estimating the (atomic) carbon reference energy relative to gaseous C_2H_2 and H_2. Standard temperature of 300 K and pressure of 1 bar are considered. Units are in eV.

	TS	ZPE
H_2	0.407	0.275
C_2H_2	0.626	0.720

Table S6: Space-group and optimized lattice constants for Pd and Au bulk reference structures.

	Spacegroup	a (Å)
Pd	225 ($Fm\bar{3}m$)	3.970
Au	225 ($Fm\bar{3}m$)	4.184

Table S7: Optimized cell parameters for (fcc) Pd 3×3×3 model structures with a variable number of intercalated carbon atoms. Cell parameters are listed in Å(lengths) and degrees (angles), volume (V) in Å^3 and formation free energy (G_{form}) in eV/atom, while referenced to gaseous C_2H_2 and H_2. The lattice expansion (in %) is calculated with respect to the cell with no carbon atoms (first row).

# C Atoms	x	y	z	α	β	γ	V	G_{form}	lattice expansion (%)
0	8.439	8.439	8.439	60°	60°	60°	424.96	0.0	0.0
1	8.492	8.492	8.492	60°	60°	60°	433.09	-0.039	0.6
2	8.545	8.537	8.537	60°	60°	60°	440.58	-0.079	1.2
3	8.6	8.574	8.574	60°	60°	60°	447.72	-0.119	1.8
4	8.636	8.629	8.623	60°	60°	60°	455.23	-0.141	2.3
5	8.672	8.681	8.664	60°	60°	60°	462.48	-0.161	2.9
6	8.715	8.725	8.694	60°	60°	60°	469.73	-0.175	3.4
7	8.79	8.76	8.724	60°	60°	60°	477.65	-0.147	4.0
8	8.86	8.784	8.764	60°	60°	60°	485.19	-0.115	4.5
9	8.917	8.807	8.807	60°	60°	60°	492.12	-0.082	5.0

Table S8: Same as in Table S7 above, but for an fcc $Pd_{0.89}Au_{0.11}$ lattice (with atomic Pd:Au ratio of 24:3 in a 3×3×3 unit cell).

# C Atoms	x	y	z	α	β	γ	V	G_{form}
0	8.479	8.479	8.475	60°	60°	60°	430.82	-0.095
1	8.534	8.534	8.53	60°	60°	60°	439.29	-0.114
2	8.589	8.589	8.583	60°	60°	60°	447.2	-0.134
3	8.646	8.646	8.612	60°	60°	60°	455.28	-0.15
4	8.687	8.687	8.693	60°	60°	60°	463.26	-0.148
5	8.728	8.776	8.754	60°	60°	60°	471.87	-0.126
6	8.814	8.814	8.83	60°	60°	60°	480.56	-0.103
7	8.885	8.885	8.9	60°	60°	60°	488.2	-0.071

Table S9: Same as in Table S7 above, but for a $Pd_{0.78}Au_{0.22}$ lattice (with atomic Pd:Au ratio of 21:6 in a 3×3×3 unit cell).

# C Atoms	x	y	z	α	β	γ	V	G_{form}
0	8.526	8.528	8.529	60°	60°	60°	438.28	-0.102
1	8.574	8.58	8.582	60°	60°	60°	446.55	-0.125
2	8.588	8.654	8.655	60°	60°	60°	455.05	-0.133
3	8.646	8.679	8.738	60°	60°	60°	463.57	-0.128
4	8.715	8.746	8.742	60°	60°	60°	472.24	-0.108
5	8.791	8.758	8.796	60°	60°	60°	481.01	-0.079

Table S10: Same as in Table S7 above, but for a $Pd_{0.67}Au_{0.33}$ lattice (with atomic Pd:Au ratio of 18:9 in a 3×3×3 unit cell).

# C Atoms	x	y	z	α	β	γ	V	G_{form}
0	8.584	8.57	8.57	60°	60°	60°	445.86	-0.111
1	8.681	8.596	8.596	60°	60°	60°	454.92	-0.1
2	8.752	8.635	8.638	60°	60°	60°	463.9	-0.087
3	8.814	8.665	8.682	60°	60°	60°	473.42	-0.072
4	8.93	8.702	8.74	60°	60°	60°	481.95	-0.036
5	9.065	8.744	8.784	60°	60°	60°	490.22	-0.001
6	9.194	8.786	8.816	60°	60°	60°	497.43	0.031
7	9.167	8.822	8.923	60°	60°	60°	506.44	0.083

Table S11: ICP-OES results of the Pd-Au LCC

Pd per gram (µg/g)	Au per gram (µg/g)	Pd per area (µg/mm^2)	Au per area (µg/mm^2)	Pd per gram (at.%)	Pd per area (at.%)
9.518	14.759	0.01865	0.028945	54.413	54.391

References

(1) Militello, M. C.; Simko, S. J. Elemental palladium by XPS. *Surface Science Spectra* **1994**, *3*, 387–394.

(2) Bancroft, G. M.; Nesbitt, H. W.; Ho, R.; Shaw, D. M.; Tse, J. S.; Biesinger, M. C. Toward a comprehensive understanding of solid-state core level XPS linewidths: Experimental and theoretical studies on the Si 2p and O 1s linewidths in silicates. *Physical Review B* **2009**, *80*, 075405.

(3) Gabasch, H.; Unterberger, W.; Hayek, K.; Klötzer, B.; Kleimenov, E.; Teschner, D.; Zafeiratos, S.; Hävecker, M.; Knop-Gericke, A.; Schlögl, R., et al. In situ XPS study of Pd(111) oxidation at elevated pressure, Part 2: Palladium oxidation in the 10^{-1} mbar range. *Surface Science* **2006**, *600*, 2980–2989.

(4) Zemlyanov, D.; Aszalos-Kiss, B.; Kleimenov, E.; Teschner, D.; Zafeiratos, S.; Hävecker, M.; Knop-Gericke, A.; Schlögl, R.; Gabasch, H.; Unterberger, W., et al. In situ XPS study of Pd(111) oxidation. Part 1: 2D oxide formation in 10^{-3} mbar O_2. *Surface Science* **2006**, *600*, 983–994.

(5) Li, Z.; Öztuna, E.; Skorupska, K.; et al., Rationally designed laterally-condensed-catalysts deliver robust activity and selectivity for ethylene production in acetylene hydrogenation. *Nature Communications* **2024**, *15*, 10660.

(6) Brundle, C. R.; Crist, B. V. X-ray photoelectron spectroscopy: A perspective on quantitation accuracy for composition analysis of homogeneous materials. *Journal of Vacuum Science and Technology A* **2020**, *38*.

(7) Brundle, C. R.; Crist, B. V.; Bagus, P. S. Accuracy limitations for composition analysis by XPS using relative peak intensities: LiF as an example. *Journal of Vacuum Science and Technology A* **2021**, *39*.

(8) Wagner, C. D.; Davis, L. E.; Zeller, M. V.; Taylor, J. A.; Raymond, R. H.; Gale, L. H. Empirical atomic sensitivity factors for quantitative analysis by electron spectroscopy for chemical analysis. *Surface and Interface Analysis* **1981**, *3*, 211–225.
